# Imputation-Based Genomic Coverage Assessments of Current Human Genotyping Arrays

**DOI:** 10.1534/g3.113.007161

**Published:** 2013-10-01

**Authors:** Sarah C. Nelson, Kimberly F. Doheny, Elizabeth W. Pugh, Jane M. Romm, Hua Ling, Cecelia A. Laurie, Sharon R. Browning, Bruce S. Weir, Cathy C. Laurie

**Affiliations:** * Department of Biostatistics, University of Washington, Seattle, Washington, 98195; † Center for Inherited Disease Research, Johns Hopkins University School of Medicine, Baltimore, Maryland, 21224

**Keywords:** genome-wide association study, genomic coverage, power, SNP microarrays

## Abstract

Microarray single-nucleotide polymorphism genotyping, combined with imputation of untyped variants, has been widely adopted as an efficient means to interrogate variation across the human genome. “Genomic coverage” is the total proportion of genomic variation captured by an array, either by direct observation or through an indirect means such as linkage disequilibrium or imputation. We have performed imputation-based genomic coverage assessments of eight current genotyping arrays that assay from ~0.3 to ~5 million variants. Coverage was determined separately in each of the four continental ancestry groups in the 1000 Genomes Project phase 1 release. We used the subset of 1000 Genomes variants present on each array to impute the remaining variants and assessed coverage based on correlation between imputed and observed allelic dosages. More than 75% of common variants (minor allele frequency > 0.05) are covered by all arrays in all groups except for African ancestry, and up to ~90% in all ancestries for the highest density arrays. In contrast, less than 40% of less common variants (0.01 < minor allele frequency < 0.05) are covered by low density arrays in all ancestries and 50–80% in high density arrays, depending on ancestry. We also calculated genome-wide power to detect variant-trait association in a case-control design, across varying sample sizes, effect sizes, and minor allele frequency ranges, and compare these array-based power estimates with a hypothetical array that would type all variants in 1000 Genomes. These imputation-based genomic coverage and power analyses are intended as a practical guide to researchers planning genetic studies.

Microarray genotyping has been widely adopted as an efficient means to interrogate variation across the human genome. Such arrays are the technological foundation of genome-wide association studies (GWAS), which have uncovered numerous genetic associations for a wide variety of traits and diseases (Manolio 2010; Visscher *et al.* 2012). The arrays used in GWAS often are designed to genotype a maximally informative set of variants that capture, or “tag,” a substantial proportion of genome-wide variation (Carlson *et al.* 2004). “Genomic coverage” refers to the total proportion of genomic variation captured by an array, either directly, via genotyping, or indirectly, through linkage disequilibrium (LD) with one or more genotyped variants (Barrett and Cardon 2006).

The determination of whether an untyped variant is “captured” by a genotyped variant can be based on one of two measures. The first measure is the maximum pairwise squared correlation (*r^2^*) between discrete allelic dosages at the untyped variant and any of the genotyped variants. Thus one way to describe genomic coverage is as the fraction of variants in a reference set having a maximum *r^2^* with array variants above a given threshold (*e.g.*, *r^2^* greater than 0.8). Genomic coverage for the first generation of SNP microarrays typically was estimated using this pairwise LD paradigm and with the HapMap Project (Frazer *et al.* 2007) as the reference set (Barrett and Cardon 2006; Li *et al.* 2008).

A second measure for determining whether an untyped variant is captured by an array involves using array variants to impute, or predict, the genotype at the untyped variant. Imputation yields a different *r^2^* from the pairwise LD *r^2^* metric described previously. “Imputation *r^2^*” is the squared correlation between the actual (discrete) allelic dosage at a genomic variant and the imputed (continuous) allelic dosage, over a defined set of samples. For a variant with two alleles *A* and *a*, the imputed dosage is calculated as 2P(*AA*) + P(*Aa*), where P(*AA*) and P(*Aa*) are posterior genotype probabilities from imputation. Under this imputation paradigm, genomic coverage can be reported as the fraction of variants in a reference set with imputation *r^2^* above a given threshold (again, usually greater than 0.8) (Hoffmann *et al.* 2011b). The reference set of variants used to determine imputation-based genomic coverage then becomes the variants present in an imputation reference panel such as the 1000 Genomes Project (Abecasis *at al*. 2012). Imputation-based genomic coverage is especially relevant to current practice in association studies, in which untyped variants in the 1000 Genomes Project are routinely imputed from a scaffold of array genotypes and tested for association with traits of interest (Marchini and Howie 2010).

An important application of *r^2^* coverage metrics is to estimate the power to detect an association when the causal locus is not observed directly. Pritchard and Przeworski (2001) found that a sample size of N for direct observation of genotypes at a causal locus gives approximately the same power as a sample size of N/*r^2^* for observed genotypes at a marker locus, where *r^2^* is the squared correlation between the discrete allele dosages at the causal and marker loci. This relationship has been used to estimate the genome-wide power to detect an association with various SNP arrays, using the maximum pairwise *r^2^* for each variant in a genome-wide reference set such as the HapMap (Li *et al.* 2008) or 1000 Genomes Projects (Lindquist *et al.* 2013). For genome-wide power, an average is taken over all variants in the reference set, using variant-specific allelic frequency and *r^2^* values (Jorgenson and Witte 2006). Here we show that the relationship between power and *r^2^* also holds when using imputation *r^2^*, the squared correlation between the discrete allelic dosage at the causal locus and the imputed allelic dosage (a continuous variable), assuming a linear relationship. We also provide genome-wide power estimates with imputation *r^2^* values, estimated for each ancestry group in a 1000 Genomes Project reference set.

Genomic coverage and power have been previously described for a number of arrays, using either HapMap (Li *et al.* 2008) or 1000 Genomes Project pilot data (Lindquist *et al.* 2013) as reference sets and using maximum pairwise *r^2^*. Updated coverage assessments are provided here using new arrays, a denser and more robust data release from the 1000 Genomes Project, and imputation-based *r^2^*. New arrays have been developed in response to both the changing needs of the genetic research community and technological advances in genotyping. While the design of the first generation of arrays was informed primarily by the HapMap Project (Frazer *et al.* 2007), array content has since expanded to include variants cataloged by the 1000 Genomes Project (Abecasis *et al.* 2012) and the Exome Sequencing Project (Tennessen *et al.* 2012), in addition to pharmacogenetic and expression quantitative trait loci markers.

Despite a growing focus on rare variants, the tagging, LD-based variant selection scheme is still of prime importance in array design. Many arrays still include a core, or “backbone,” of GWAS markers selected using the same principles as the first generation of arrays. Each of the major commercial vendors has recently released (Affymetrix 2012; Illumina 2012) products with a foundation of approximately 240,000 GWAS tagging markers: the Illumina (www.illumina.com) Infinium HumanCore and the Affymetrix (www.affymetrix.com) Axiom Biobank. Although vendors frequently provide genomic coverage estimates in product documentation, the methods used are often different and/or not well-described, making it difficult to objectively compare across arrays. Here we have used a consistent, imputation-based approach to evaluate genomic coverage and power across eight different genotyping arrays. These analyses are intended to function as a practical guide to researchers who are planning genetic studies. The results may be used to compare the cost efficiency of commercially available arrays, to assess the power to detect trait associations using imputed allelic dosages, and to design custom arrays. They also provide an evaluation of the accuracy of imputation for different categories of minor allele frequency (MAF).

## Materials and Methods

### Genomic coverage

**Figure 1:**
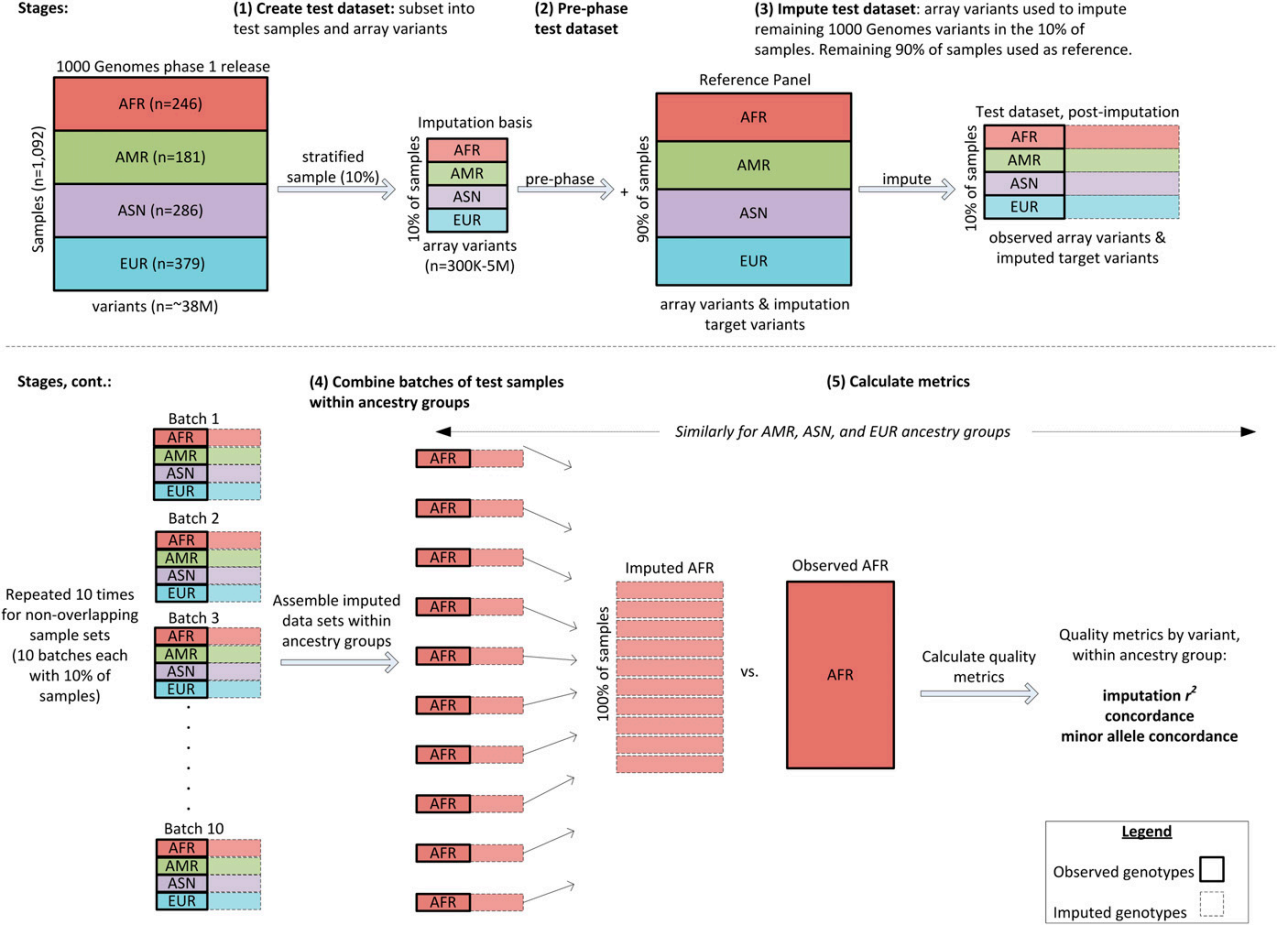
Study design. Schematic of the method used to assess genomic coverage of each array. Throughout the diagram, bold solid lines around boxes indicate observed genotypes (*i.e.*, variant calls from the 1000 Genomes Project phase 1 integrated release, version 3), whereas dashed lines indicate imputed genotypes.

We assessed genomic coverage with the design outlined in Figure 1 by using the publicly available 1000 Genomes Project (Abecasis *et al.* 2012) phase 1 integrated variant set, in variant call format (VCF, available at ftp://ftp.1000genomes.ebi.ac.uk/vol1/ftp/release/20110521/), comprising 1,092 samples from 14 populations (Table 1). For each array in turn, we selected 1000 Genomes Project variants from the VCF file having the same position as variants on the array. The array variants were first pre-phased with SHAPEIT2 software (Delaneau *et al.* 2013) and then used to impute the remaining 1000 Genomes variants, with IMPUTE2 software (Howie *et al.* 2011). The 1092 samples were randomly divided into 10 batches (balancing across populations) for these pre-phasing and imputation steps, with the remaining samples serving as a worldwide imputation reference panel. After imputation, the ten batches were combined to calculate accuracy metrics within each ancestry group. This approach is similar to the leave-one-out strategy used by Lindquist *et al.* (2013) to estimate imputation-based genomic coverage.

**Table 1:** Samples in the 1000 Genomes Project phase I integrated variant set

Full Population Name	Abbreviation	No. Samples
African Ancestry in Southwest US	ASW	61
Luhya in Webuye, Kenya	LWK	97
Yoruba in Ibadan, Nigeria	YRI	88
**Total African ancestry**	**AFR**	**246**
Colombian in Medellin, Colombia	CLM	60
Mexican Ancestry in Los Angeles, CA	MXL	66
Puerto Rican in Puerto Rico	PUR	55
**Total American ancestry**	**AMR**	**181**
Han Chinese in Beijing, China	CHB	97
Han Chinese South, China	CHS	100
Japanese in Tokyo, Japan	JPT	89
**Total Asian ancestry**	**ASN**	**286**
Utah residents (CEPH) with Northern and Western European ancestry	CEU	85
Toscani in Italia	TSI	98
British in England and Scotland	GBR	89
Finnish in Finland	FIN	93
Iberian populations in Spain	IBS	14
**Total European ancestry**	**EUR**	**379**

The Project has grouped these 1092 samples into four ancestry groups representing the “predominant component of ancestry”: African (AFR), American (AMR), Asian (ASN), and European (EUR) (Abecasis *et al.* 2012).

To assess imputation accuracy and by extension genomic coverage, we compared imputed results at all the nonarray variants to observed genotypes from the initial VCF files. These comparisons were performed separately in the four different ancestry groups (African, “AFR;” American, “AMR;” Asian, “ASN;” and European, “EUR”) and restricted to variants with at least two copies of the minor allele. For each imputed variant we calculated three metrics: (1) the squared correlation between observed and imputed allelic dosage, which we call “imputation *r^2^*”; (2) the concordance between observed and most likely imputed genotype, the “genotype concordance”; and (3) the concordance between observed and most likely imputed genotype, when at least one of those two genotypes contains one or two copies of the minor allele, which we call “minor allele (MA) concordance.” Array (observed) variants are included in these metrics summaries and are given imputation *r^2^*, genotype concordance, and MA concordance values of 1. A more detailed account of data preparation, pre-phasing, imputation, and metrics calculation is available in Supporting Information, File S1.

We focus on imputation *r^2^* as the primary coverage metric mainly due to its simple relationship to power, in addition to the following advantages: (1) precedent in the literature for evaluating both imputation accuracy (Howie *et al.* 2011, 2012; Delaneau *et al.* 2013) and array coverage (Hoffmann *et al.* 2011a,b; Lindquist *et al.* 2013); (2) less sensitivity to allele frequency than concordance; (3) similarity to information metrics commonly reported by imputation software (for a review, see Marchini and Howie 2010); and (4) incorporation of imputation uncertainty by using expected allelic dosage rather than most likely genotype. However, one important caveat is that *r^2^* has high variance at low MAF (Evangelou and Ioannidis 2013). Overall genotype concordance is also a widely used metric that is easily interpretable, although it ignores imputation uncertainty and is very sensitive to allele frequency, as low MAF variants may yield high concordance purely by chance (Lin *et al.* 2010). MA concordance helps correct for sensitivity to MAF by not counting correctly imputed major homozygous genotypes. Thus, although we have used imputation *r^2^* as our primary coverage metric and the basis for the power analyses, we also provide coverage in terms of MA concordance and concordance to enable downstream users the flexibility to focus on one of these alternative metrics.

### Power analyses

The power to detect a variant-trait association was calculated for a case-control study under the assumption of an additive genetic model, using the following parameters, where *A* is the risk allele and *a* is the alternate allele: genotype relative risk (GRR, of *Aa* relative to *aa*) from 1.1 to 1.4 in increments of 0.1; disease prevalence (K) of 0.05; frequency of the risk allele (p) assumed in each case to be the minor allele; the sample size (N cases and N controls) from N = 1000 to 10,000 in increments of 50; and a significance level of 5 × 10^−8^ (appropriate for genome-wide testing). The assumption of an additive model is based on how imputed dosages are used in association tests, and the range of GRR is based on a summary of effect estimates from genome-wide association studies (Lindquist *et al.* 2013). Power was calculated for an allelic association test (χ^2^ with 1 degree of freedom) using the non-centrality parameter in the Appendix. Sample size was N for observed (array) variants and N/(imputation *r^2^*) for imputed variants. A genome-wide power estimate was obtained for each array and parameter set by averaging over all variants in a reference set, as indicated by Jorgenson and Witte (2006). Here, the reference set consists of 1000 Genomes variants with at least two copies of the minor allele (or a subset thereof). In principle, power would be calculated for each individual variant (using its individual MAF and *r^2^* values) and averaged over all variants in the set. In practice, to reduce computation, we binned all autosomal variants by a combination of MAF and *r^2^* values, calculated power for the mean values of MAF and *r^2^* within each bin, and took the mean over bins weighted by the fraction of variants in each bin, as in Li *et al.* (2008). Binning for MAF was from 0 to 0.05 in increments of 0.005, from 0.05 to 0.10 in increments of 0.01, and from 0.1 to 0.5 in increments of 0.05. Binning for *r^2^* was from 0 to 1 in increments of 0.05.

## Results and Discussion

We have assessed imputation-based genomic coverage for eight commercially available genotyping arrays using the four ancestry groups in the 1000 Genomes Project phase 1 release. The number of assays and unique genomic positions assayed by each array are summarized in Table 2. Only those array variants also found in the 1000 Genomes phase 1 integrated variant set were able to inform the imputation and thus contribute to these genomic coverage estimates. To see how this fact may differentially impact arrays, in Table 2 we also report (1) the percent overlap with 1000 Genomes at any MAF and (2) the percent overlap with 1000 Genomes at the requisite MAF to be included in the imputation (*i.e.*, at least two copies of the minor allele observed in any one of the four ancestry groups). As may be expected, the overlap with 1000 Genomes for arrays with a high proportion of rare and exome variants is less than for arrays with less content devoted to rare and exome variants. The possible impact of the differential 1000 Genomes overlap across arrays on the estimated genomic coverage appears to be small (see File S1).

**Table 2:** Array summaries

Company	Array	Product Information*^a^*	No. Assays	No. Positions	Percent Overlap with 1000 Genomes	Percent Overlap with 1000 Genomes, MAF Filtered
Illumina	HumanCore	12v1-0, A	296,720	296,677	88.2	87.8
Illumina	HumanCore+Exome	12v1-0, B	535,743	528,484	76.3	69.3
Affymetrix	Axiom Biobank	na33	716,836	645,209	73.6	67.7
Illumina	OmniExpress	12v1, H	727,413	727,410	98.8	98.8
Affymetrix	Axiom World Array 4	na32	841,602	814,831	98.6	98.5
Illumina	Omni2.5M	8v1, A	2,368,218	2,362,580	93.2	93.0
Illumina	Omni2.5M+Exome	8v1, A	2,556,812	2,513,578	91.5	89.9
Illumina	Omni5M	4v1, C	4,285,657	4,279,793	94.4	93.2

Summaries of each array included in these genomic coverage analyses, restricted to assays mapped to chromosomes 1 through 22 and the non-pseudoautosomal portion of the X chromosome. For each array, the columns give (1) the company manufacturing the array; (2) the array name; (3) additional product information; (4) the total number of assays; (5) the total number of unique map locations represented by those assays; (6) the percent overlap with 1000 Genomes phase 1 integrated variant set, at any frequency; and (7) the percent overlap with 1000 Genomes phase 1 integrated variant set, with a minimum of two copies of the minor allele seen in at least one of the four ancestry groups. The counts in (5) are less than (4) when there is more than one assay/feature for a given genomic position. The percentages given in (6) and (7) are with a denominator of unique positions, rather than unique assays. MAF, minor allele frequency.

a Product information. For Illumina arrays, number of samples (*e.g.*, 4, 8, or 12), version of the array (*e.g.*, “v1”), and version of the array manifest file (.bpm, *e.g.*, “A” or “B”). For Affymetrix arrays, the NetAffx release number.

**Figure 2:**
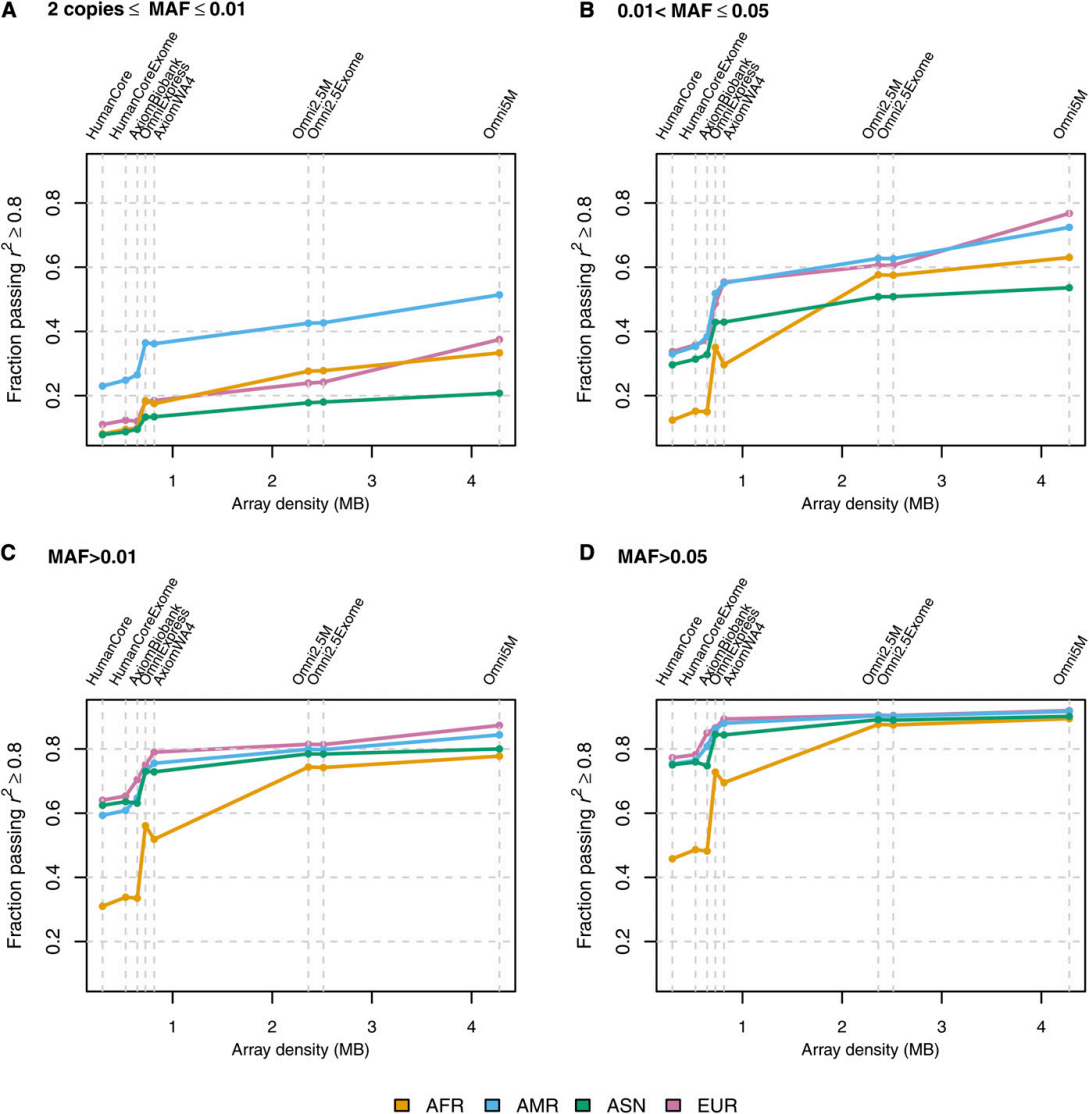
Fraction of variants passing an imputation *r^2^* threshold of 0.8, by MAF bin and ancestry group. The imputation *r^2^* metric plotted here is the squared correlation between imputed and observed allelic dosage in the samples comprising the ancestry group. The y-axis is the proportion of variants (imputed and observed) with imputation *r^2^* ≥ 0.8, restricted to variants with at least two copies of the minor allele in the given ancestry group. The x-axis position of each array corresponds to the number of unique positions assayed by that array (see Table 2, column 5). Thus. the order of the arrays on each axis is as follows: HumanCore, HumanCore+Exome, Axiom Biobank, OmniExpress, Axiom World Array 4, Omni2.5M, Omni2.5M+Exome, and Omni5M. Panel (A) is for variants with at least two copies of the minor allele and MAF ≤ 0.01, (B) for 0.01 < MAF ≤ 0.05, (C) for MAF > 0.01, and (D) for MAF > 0.05.

**Figure 3:**
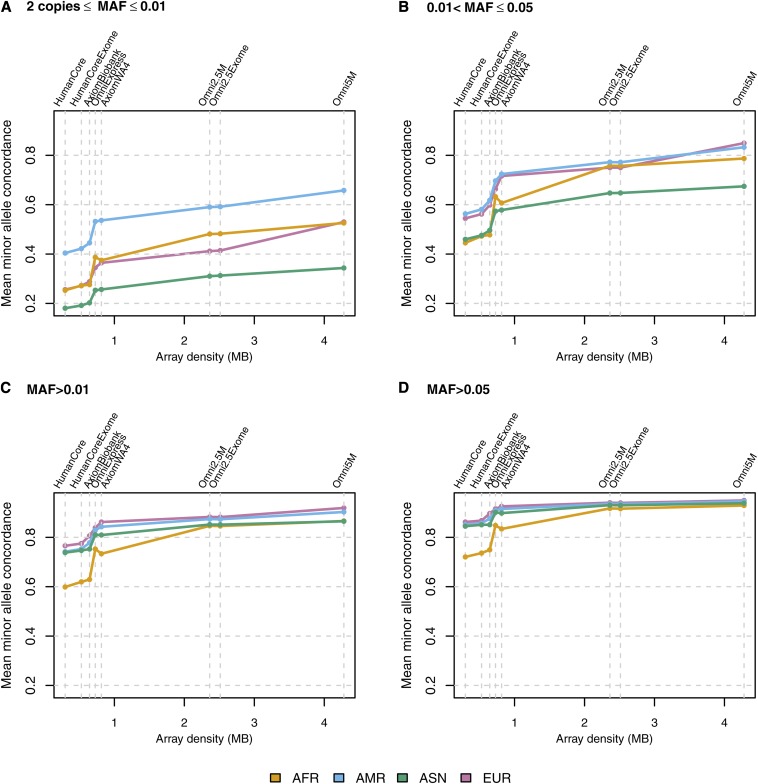
Mean MA concordance, by MAF bin and ancestry group. The y-axis values are mean MA concordance in samples comprising the given ancestry group. MA concordance is defined as the concordance (percent agreement) between observed and most likely imputed genotype, when at least one of those two genotypes contains one or two copies of the minor allele. Variants were restricted to those with at least two copies of the minor allele in the given ancestry group. The x-axis position of each array corresponds to the number of unique positions assayed by that array (see Table 2, column 5). Thus the order of the arrays on each axis is as follows: HumanCore, HumanCore+Exome, Axiom Biobank, OmniExpress, Axiom World Array 4, Omni2.5M, Omni2.5M+Exome, and Omni5M. (A) Variants with at least two copies of the minor allele and MAF ≤ 0.01, (B) for 0.01 < MAF ≤ 0.05, (C) for MAF > 0.01, and (D) for MAF > 0.05.

Figure 2 shows the fraction of variants passing an imputation *r^2^* threshold of 0.8, by MAF bin and ancestry group, whereas Figure 3 shows the mean MA concordance. Genomic coverage assessments commonly use the MAF groupings of > 0.01 and > 0.05, which we have shown in Figure 2 and Figure 3, C and D, respectively. All the data plotted in Figure 2 and Figure 3 are presented numerically in Table 3 and Table 4, which also include tabular summaries of mean imputation *r^2^* and genotype concordance and the counts of variants in each MAF bin, as this count differs by ancestry group. Line plots of mean imputation *r^2^* and mean genotype concordance are available in Figure S1 and Figure S2. Plots organized by ancestry panel rather than by metric are available for AFR in Figure S3, AMR in Figure S4, ASN in Figure S5, and EUR in Figure S6.

**Table 3:** Genome-wide coverage estimates for all eight arrays, in AFR and AMR ancestry groups

Ancestry Group		AFR (n = 246)				AMR (n = 181)			
MAF bin		0.4%<MAF≤ 1%	1%<MAF≤ 5%	MAF>1%	MAF>5%	0.6%<MAF≤1%	1%<MAF≤5%	MAF>1%	MAF>5%
Number of variants		5,567,596	7,515,313	16,995,351	9,480,038	3,783,595	4,245,247	11,211,369	6,966,122
Array	Metric								
HumanCore	*r^2^* ≥ 0.8 (%)	8.1%	12.4%	31.0%	45.8%	23.0%	33.0%	59.3%	75.4%
	*r^2^* (mean)	0.275	0.447	0.588	0.699	0.446	0.576	0.741	0.842
	MA conc (mean)	0.253	0.445	0.599	0.721	0.404	0.563	0.742	0.851
	geno conc (mean)	0.989	0.968	0.934	0.907	0.990	0.976	0.958	0.946
HumanCore + Exome	*r^2^* ≥ 0.8 (%)	9.5%	15.1%	33.8%	48.6%	24.8%	35.3%	60.9%	76.4%
	*r^2^* (mean)	0.296	0.476	0.610	0.717	0.463	0.594	0.752	0.849
	MA conc (mean)	0.273	0.473	0.620	0.736	0.422	0.581	0.753	0.857
	geno conc (mean)	0.989	0.969	0.938	0.912	0.990	0.977	0.959	0.949
Axiom Biobank	*r^2^* ≥ 0.8 (%)	9.9%	15.0%	33.5%	48.2%	26.5%	38.4%	64.7%	80.8%
	*r^2^* (mean)	0.299	0.482	0.622	0.733	0.488	0.633	0.780	0.870
	MA conc (mean)	0.276	0.478	0.629	0.749	0.445	0.618	0.778	0.876
	geno conc (mean)	0.989	0.970	0.939	0.914	0.991	0.980	0.964	0.955
OmniExpress	*r^2^* ≥ 0.8 (%)	18.4%	35.0%	56.1%	72.8%	36.4%	51.8%	73.1%	86.0%
	*r^2^* (mean)	0.415	0.644	0.754	0.841	0.569	0.708	0.830	0.904
	MA conc (mean)	0.387	0.632	0.753	0.848	0.532	0.697	0.829	0.910
	geno conc (mean)	0.991	0.980	0.964	0.951	0.992	0.984	0.974	0.967
Axiom World Array 4	*r^2^* ≥ 0.8 (%)	17.5%	29.6%	51.9%	69.5%	36.2%	55.1%	75.5%	88.0%
	*r^2^* (mean)	0.401	0.619	0.735	0.828	0.577	0.737	0.845	0.910
	MA conc (mean)	0.375	0.607	0.734	0.834	0.536	0.724	0.843	0.915
	geno conc (mean)	0.991	0.979	0.961	0.947	0.993	0.986	0.975	0.968
Omni2.5M	*r^2^* ≥ 0.8 (%)	27.6%	57.6%	74.4%	87.7%	42.6%	62.7%	79.9%	90.3%
	*r^2^* (mean)	0.509	0.769	0.850	0.914	0.625	0.781	0.874	0.931
	MA conc (mean)	0.481	0.758	0.847	0.917	0.590	0.772	0.874	0.936
	geno conc (mean)	0.993	0.988	0.979	0.973	0.993	0.989	0.981	0.976
Omni2.5M + Exome	*r^2^* ≥ 0.8 (%)	27.8%	57.5%	74.2%	87.5%	42.7%	62.7%	79.8%	90.2%
	*r^2^* (mean)	0.510	0.769	0.849	0.912	0.626	0.780	0.874	0.930
	MA conc (mean)	0.482	0.757	0.846	0.916	0.592	0.772	0.873	0.935
	geno conc (mean)	0.993	0.988	0.979	0.972	0.993	0.989	0.981	0.976
Omni5M	*r^2^* ≥ 0.8 (%)	33.3%	63.0%	77.8%	89.4%	51.4%	72.4%	84.4%	91.7%
	*r^2^* (mean)	0.553	0.797	0.869	0.926	0.690	0.838	0.902	0.941
	MA conc (mean)	0.526	0.787	0.866	0.929	0.658	0.833	0.903	0.945
	geno conc (mean)	0.993	0.989	0.982	0.976	0.995	0.992	0.984	0.980

Four metrics are presented for each array: fraction of variants with imputation *r^2^* ≥ 0.8; mean imputation *r^2^*; mean minor allele concordance (“MA conc”); and mean genotype concordance (“geno conc”); separately by ancestry group (AFR, AMR) and MAF bin. Note the lower bound of MAF in the first MAF bin differs across ancestry groups. This is because we required at least two copies of the minor allele in each panel in order to contribute to these metrics summaries, and there are differing numbers of samples in each group. AFR, African, AMR, American, MAF, minor allele frequency.

**Table 4:** Genome-wide coverage estimates for all eight arrays, in ASN and EUR ancestry groups

Ancestry group		ASN (n = 286)				EUR (n = 379)			
MAF bin		0.4%<MAF≤ 1%	1%<MAF≤5%	MAF>1%	MAF>5%	0.3%<MAF≤1%	1%<MAF≤5%	MAF>1%	MAF>5%
Number of variants		3,004,524	2,385,094	8,606,085	6,220,991	3,991,091	2,944,222	9,705,585	6,761,363
Array	Metric								
HumanCore	*r^2^* ≥ 0.8 (%)	7.8%	29.6%	62.4%	75.0%	11.0%	33.7%	64.1%	77.3%
	*r^2^* (mean)	0.193	0.455	0.726	0.830	0.269	0.544	0.758	0.851
	MA conc (mean)	0.180	0.460	0.738	0.845	0.256	0.545	0.766	0.862
	geno conc (mean)	0.988	0.968	0.948	0.941	0.991	0.974	0.957	0.950
HumanCore + Exome	*r^2^* ≥ 0.8 (%)	8.7%	31.4%	63.6%	75.9%	12.3%	35.8%	65.3%	78.2%
	*r^2^* (mean)	0.205	0.471	0.735	0.836	0.285	0.562	0.767	0.857
	MA conc (mean)	0.192	0.476	0.747	0.851	0.271	0.562	0.775	0.868
	geno conc (mean)	0.988	0.969	0.950	0.943	0.991	0.975	0.959	0.952
Axiom Biobank	*r^2^* ≥ 0.8 (%)	9.5%	32.8%	63.1%	74.8%	12.0%	37.1%	70.5%	85.0%
	*r^2^* (mean)	0.215	0.492	0.742	0.839	0.303	0.603	0.803	0.890
	MA conc (mean)	0.203	0.496	0.753	0.851	0.288	0.598	0.806	0.897
	geno conc (mean)	0.989	0.970	0.951	0.943	0.991	0.978	0.966	0.962
OmniExpress	*r^2^* ≥ 0.8 (%)	13.3%	42.9%	73.0%	84.5%	18.1%	48.7%	75.1%	86.6%
	*r^2^* (mean)	0.267	0.570	0.802	0.892	0.359	0.665	0.834	0.908
	MA conc (mean)	0.253	0.574	0.810	0.901	0.346	0.663	0.839	0.915
	geno conc (mean)	0.990	0.976	0.966	0.962	0.992	0.981	0.973	0.969
Axiom World Array 4	*r^2^* ≥ 0.8 (%)	13.4%	42.9%	72.9%	84.4%	18.5%	55.4%	79.0%	89.3%
	*r^2^* (mean)	0.269	0.574	0.802	0.889	0.380	0.720	0.859	0.920
	MA conc (mean)	0.257	0.578	0.809	0.898	0.364	0.716	0.862	0.926
	geno conc (mean)	0.989	0.976	0.965	0.961	0.992	0.985	0.976	0.971
Omni2.5M	*r^2^* ≥ 0.8 (%)	17.8%	50.8%	78.5%	89.1%	23.9%	60.6%	81.5%	90.5%
	*r^2^* (mean)	0.324	0.643	0.846	0.923	0.426	0.752	0.879	0.935
	MA conc (mean)	0.310	0.647	0.852	0.930	0.412	0.750	0.883	0.940
	geno conc (mean)	0.990	0.980	0.975	0.974	0.993	0.986	0.980	0.977
Omni2.5M + Exome	*r^2^* ≥ 0.8 (%)	18.0%	50.8%	78.4%	89.0%	24.2%	60.6%	81.4%	90.4%
	*r^2^* (mean)	0.326	0.643	0.845	0.923	0.428	0.752	0.879	0.934
	MA conc (mean)	0.313	0.648	0.851	0.930	0.414	0.750	0.882	0.940
	geno conc (mean)	0.990	0.980	0.975	0.973	0.993	0.986	0.980	0.977
Omni5M	*r^2^* ≥ 0.8 (%)	20.7%	53.6%	80.0%	90.2%	37.5%	76.8%	87.3%	91.9%
	*r^2^* (mean)	0.357	0.670	0.859	0.932	0.545	0.849	0.916	0.944
	MA conc (mean)	0.344	0.674	0.865	0.938	0.530	0.849	0.919	0.949
	geno conc (mean)	0.991	0.982	0.978	0.977	0.995	0.991	0.984	0.980

Four metrics are presented for each array: fraction of variants with imputation *r^2^* ≥ 0.8; mean imputation *r^2^*; mean minor allele concordance (“MA conc”); and mean genotype concordance (“geno conc”); separately by ancestry group (ASN, EUR) and MAF bin. Note the lower bound of MAF in the first MAF bin differs across ancestry groups. This is because we required at least two copies of the minor allele in each panel to contribute to these metrics summaries, and there are differing numbers of samples in each group. ASN, Asian, EUR, European, MAF, minimum allele frequency.

As one might expect, coverage generally improves with increasing array density, regardless of either ancestry group or MAF bin. Common variants (MAF > 0.05) are well-covered by all the arrays in the AMR, ASN, and EUR ancestry groups. The percentage of common variants with imputation *r^2^* ≥ 0.8 is greater than 75% in these three ancestry groups, and up to ~90% in all four ancestry groups for the highest density arrays. The most dramatic increase in coverage occurs for common variants in the AFR group: the fraction of common variants passing the imputation *r^2^* threshold of 0.8 increases by almost 40% from the least to most dense array. This finding is likely explained by the genetic diversity and lower LD characteristic of present day African ancestry populations compared to the other ancestry groups (Wall *et al.* 2008).

However, coverage at common variants eventually levels off with increasing array density, a phenomenon of diminishing returns previously observed by Barrett and Cardon (2006). The array density at which this occurs differs by ancestry group. For AMR, ASN, and EUR, leveling off begins at the OmniExpress, whereas for AFR, coverage continues to improve up until the Omni2.5M. This plateau in coverage may be in part due to the limited size of the reference sample, and in part due to the underlying structure of the genome being such that some regions have insufficient LD to accurately impute variants, regardless of MAF. Furthermore, even with high density arrays, there may still be some regions that are sparsely covered and therefore difficult to impute.

Also as expected, less common variation is not as well covered as common variation. The percentage of variants with MAF between 0.01 and 0.05 and an imputation *r^2^* ≥ 0.8 is substantially lower than for MAF greater than 0.05: less than 40% for low density arrays in all ancestries and 50–80% in high density arrays, depending on ancestry. In all ancestry groups there is some improvement in coverage of less common variants when moving from the Omni2.5M to the Omni5M array, most notably in EUR (61% *vs.* 77% with *r^2^* ≥ 0.8 with Omni2.5M and Omni5M, respectively). Rare (MAF < 0.01) variants are generally not well imputed by either low or high density arrays. Imputation accuracy for rare variants is greatest in the AMR ancestry group, which might be a consequence of the lower genetic diversity and greater LD in Native Americans than in other ancestry groups, due to a recent population bottleneck associated with human migration into the Americas (Wall *et al.* 2011). Imputation quality at rare variants may be improved by replacing pre-phased imputation with the traditional haplotype sampling approach, although the latter is more computationally intensive (Howie *et al.* 2012).

All coverage plots are essentially monotonic, with the exception of the transition from the OmniExpress to the Axiom World Array 4 in the AFR ancestry group. This array was designed for use in Latinos and involved preferential selection of variants in European and Native American ancestries (Hoffmann *et al.* 2011b), which might explain the lower coverage in the AFR group. We also note that the addition of the exome content has little effect on coverage and accuracy, particularly for the Omni2.5M. The HumanCore+Exome, however, shows slight improvement over the HumanCore alone for lower MAF variants, most likely because the addition of the exome content affords a greater relative increase in density for the HumanCore than for the Omni2.5M. Although exome content contributes little to overall genomic coverage, it clearly has additional value when directly observed, in that exomic variants are more likely to be functional than non-exomic variants. Furthermore, this value comes at relatively low additional cost.

### Genome-wide power

Previous studies of genome-wide power using arrays have utilized a finding that the sample size required to achieve a given power is N/*r^2^* when using a tag marker compared with N when using the causal locus genotypes, where *r^2^* is the squared correlation between the discrete allelic dosages at each locus (Pritchard and Przeworski 2001). The Appendix shows that this relationship also holds under an additive genetic model when using a continuous imputed dosage rather than the discrete dosage of the causal locus. Therefore, we used imputation *r^2^* for estimating the genome-wide power to detect an association.

**Figure 4:**
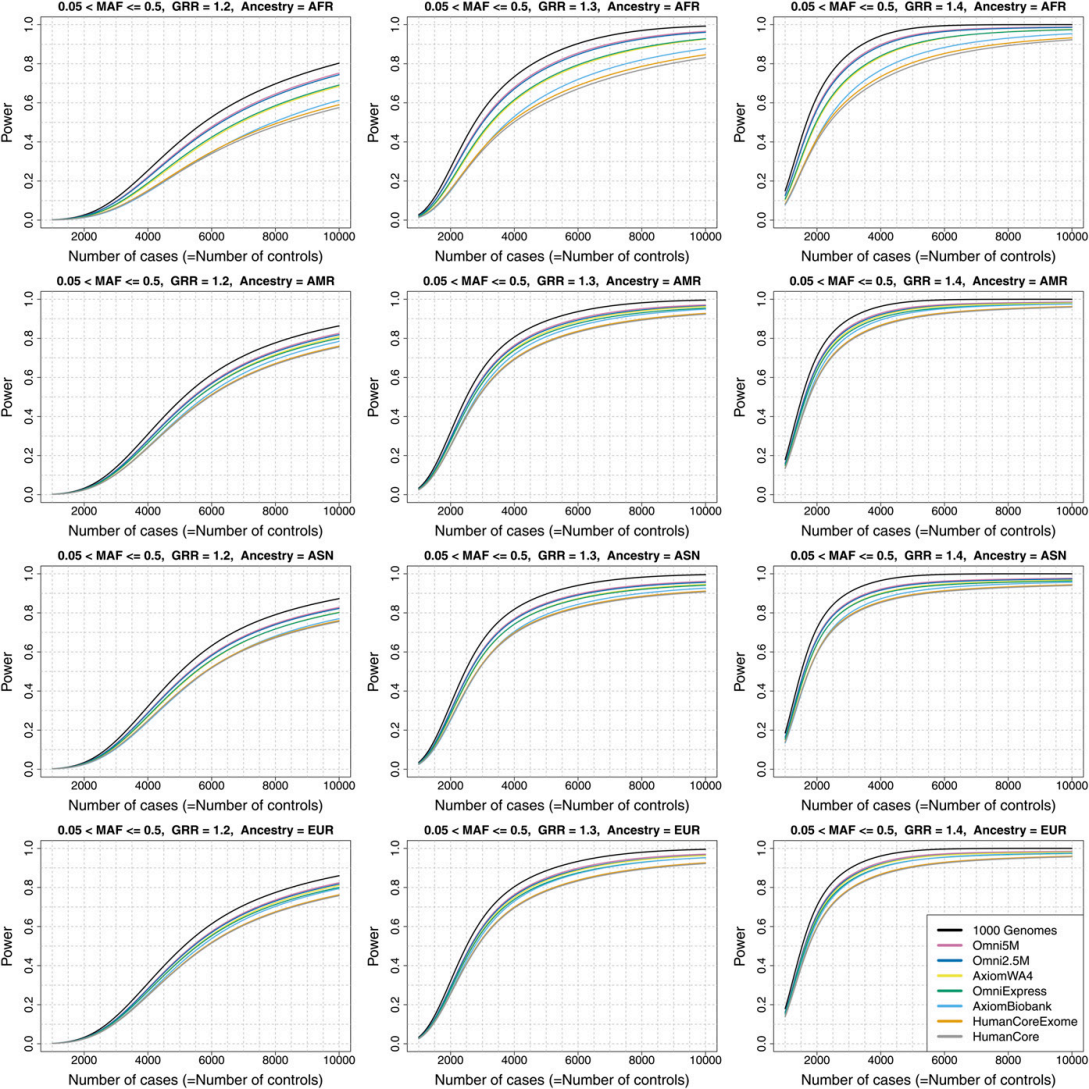
Genome-wide power estimates for GRR values of 1.2, 1.3, and 1.4, for common autosomal variants (MAF > 0.05). The array Omni2.5+Exome is not shown in these plots because it is indistinguishable at this resolution from the Omni2.5M array. In the legend, “1000 Genomes” refers to a hypothetical array in which all variants in the 1000 Genomes dataset would be typed.

**Figure 5:**
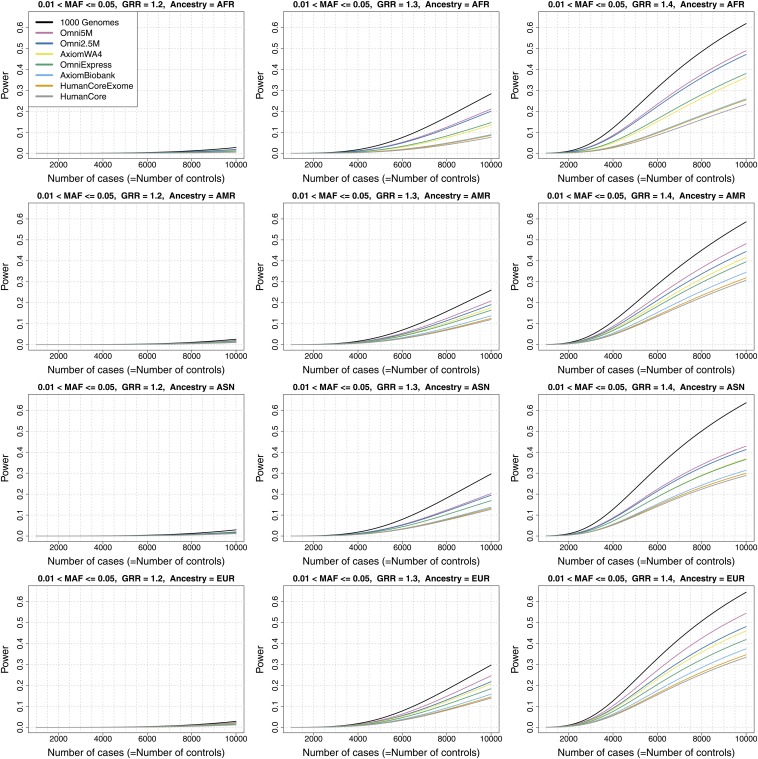
Genome-wide power estimates for GRR values of 1.2, 1.3, and 1.4, for less common autosomal variants (0.01 < MAF ≤ 0.05). The array Omni2.5+Exome is not shown in these plots because it is indistinguishable at this resolution from the Omni2.5M array. In the legend, “1000 Genomes” refers to a hypothetical array in which all variants in the 1000 Genomes dataset would be typed.

Genome-wide power estimates for GRR values of 1.2, 1.3 and 1.4 are displayed in Figure 4 for MAF > 0.05 (common variants), Figure 5 for 0.01 < MAF ≤ 0.05 (less common), Figure S7 for MAF > 0.01 and Figure S8 for all variants with at least two copies of the minor allele within an ancestry group. Figure S9 displays results for GRR = 1.1 for each of the four MAF categories. The power curves show a sigmoidal relationship between power and sample size for all arrays, including the hypothetical 1000 Genomes array (“1000 Genomes”), which is equivalent to direct observation of all 1000 Genomes Project variants passing the ancestry-specific MAF filters (*i.e.*, at least two copies of the minor allele observed in the given ancestry group) and thus does not involve uncertainty introduced by imputation. Power is generally low for the range of parameters considered here (even for “1000 Genomes” array), except at the upper end of genetic effect and sample sizes. This is particularly true for the less common variants, where power is generally < 50% even at the high end of the parameter space for the actual arrays and up to ~60% for the “1000 Genomes” array.

The cost efficiency of achieving equal power can be compared among array choices using these power estimates. For example, for the variant set with MAF > 0.05 and GRR = 1.3, achieving 50% power requires N = 3000 for the Omni2.5M array and N = 4000 for the HumanCore array in the African ancestry group. A power of 80% requires a larger sample size difference (5300 *vs.* 8900). Generally, the array differences required to achieve a set power are notably larger in regions where the power curves plateau. One array is more cost-efficient than another when the ratio of cost per sample is less than the ratio of sample sizes required to achieve equal power. Of course, this evaluation assumes that the extra samples required are available and that the main interest of the study is to detect effects characterized by the parameters used in the power calculation. All of the data used to generate the power plots are available for download at http://jhir.library.jhu.edu/handle/1774.2/36508, so that users can select parameter sets appropriate to their project goals. We also provide the number of variants per MAF and *r^2^* combinations within each ancestry group so that users can readily calculate power using parameters beyond the set provided here.

As noted previously in a similar analysis by Lindquist *et al.* (2013), increasing sample size can benefit genome-wide power more than increasing array density even up to full genotyping of the complete 1000 Genomes variant set. For example, with GRR = 1.3 and MAF > 0.05 in African ancestry, power is 26.7% for the “1000 Genomes” array with N = 2000, whereas doubling the sample size and using the HumanCore array brings power up to 50.2%. Lindquist *et al.* (2013) focused their analysis on variants with characteristics similar to those found in previous GWAS (odd ratios mainly in the range of 1.2−1.3 and MAF > 0.10). They concluded that only about one fifth of such variants have been detected with existing GWAS and that the potential for increasing this fraction is greater by increasing sample size than by increasing genomic coverage. Our results support this conclusion (using somewhat different methods and arrays), given the current situation where low density arrays such as the Illumina HumanCore and Affymetrix BioBank are much lower in cost than genomic sequencing or even high density arrays such as the Omni2.5M. However, we also note their caveat that increasing sample size significantly is not an option for many diseases.

For rare variants, power is low, even for complete coverage and very large sample sizes. Therefore, analysis of rare variants generally involves aggregation strategies to achieve reasonable power to detect an effect (Li and Leal 2008). The value of low density arrays in this context appears to be low because rare variants are not well imputed and aggregate tests are sensitive to genotype errors (Powers *et al.* 2011). Therefore, direct detection of rare variants using genomic sequencing, high density arrays and/or arrays supplemented with selected variants of interest (such as exome content) are likely to be required for making significant progress in detecting trait variation caused by rare variants.

In addition to planning experiments with existing arrays, our genomic coverage results can be used to design new genotyping arrays. For example, one might wish to design an array focused on specific candidate variants or regions, while also providing a backbone for genome-wide imputation, such as the existing HumanCore or Biobank variant set. In that case, one might begin with the backbone and then add candidate variants that are not already well imputed by the backbone variants in the target ancestry group(s). For this purpose, we also provide the individual imputation *r^2^* values for all 1000 Genomes variants from imputation using each of the arrays considered here (available at http://jhir.library.jhu.edu/handle/1774.2/36508).

In conclusion, these imputation-based genomic coverage and power analyses are intended as a practical guide to researchers planning genetic studies. Deciding among available arrays is often a delicate balance between scientific aims, monetary resources, and genotyping timeframe (*i.e.*, which arrays are currently in use). Our analysis has the advantage of consistently applying the same approach to evaluate several different arrays from different manufacturers. There are, however, some limitations to this study design that may result in either over- or underestimated genomic coverage. First, the genomic coverage reported here might be over estimated by imputing subsets of 1000 Genomes samples using the remaining samples as reference, as the haplotypes are likely to match better than in real world applications where the samples to be imputed are likely from different source populations than the reference. This advantage may be offset to some degree by using a smaller reference panel than is available for independent study populations, although this effect is likely to be small because the reference panel used here is approximately 90% of the full 1000 Genomes phase 1 reference panel. Second, coverage may be underestimated by having pre-phased each batch separately, as small sample sizes may negatively impact phasing accuracy (Browning and Browning 2011). An increased sample size during pre-phasing may disproportionally improve coverage in situations where phasing accuracy is already a challenge, such as rare variants and in samples with high haplotype diversity (*e.g.*, African ancestry). Finally, some of the arrays assay a substantial number of variants that are not in the 1000 Genomes phase 1 release (see Table 2). These omissions would tend to underestimate genomic coverage, although probably not significantly, since they are expected to consist mainly of rare variants due to the 1000 Genomes Project’s identification of 98% of SNPs with frequency > 0.01 in related populations (Abecasis *et al.* 2012). Despite these caveats (discussed further in File S1), the 1000 Genomes data set is clearly the best resource available for evaluating complete genomic coverage in multiple ancestry groups. We expect the results presented here to be useful in planning studies with the current generation of human genotyping arrays.

## Supplementary Material

Supporting Information

## Appendix

Here we show that the effect of imputation uncertainty on the power to detect a trait association is to increase the required sample size from *n* to *n*/*ρ*^2^, where *ρ*^2^ is the squared correlation between true and imputed allelic dosage.

### Notation

Suppose a trait locus **T** has alleles *T*, *t* with population frequencies *p_T_*, *p_t_* and that variable *G* represents the contributions of **T** to the trait. The values *G_TT_*, *G_Tt_*, and *G_tt_* are the probabilities of an individual having the disease under study. The mean of *G* is the population prevalence of the disease, *μ_G_*, and under Hardy Weinberg equilibrium (HWE), this is

$$\mu_{G}=p_{T}^{2}G_{TT}+2p_{T}p_{t}G_{Tt}+p_{t}^{2}G_{tt}$$

Also under HWE, the genetic variance of the *G*’s can be written as follows:

$$\mathrm{Var}\left(G\right)=\sigma_{A_{G}}^{2}+\sigma_{D_{G}}^{2}$$

where the additive and dominance variance components, $\sigma_{A_{G}}^{2}$ and $\sigma_{D_{G}}^{2}$ , respectively, are given by

$$\begin{array}{l} \sigma_{A_{G}}^{2}=2p_{T}p_{t}{\left[p_{T}\left(G_{TT}-G_{Tt}\right)+p_{t}\left(G_{Tt}-G_{tt}\right)\right]}^{2}\\ \sigma_{D_{G}}^{2}=p_{T}^{2}p_{t}^{2}{\left(G_{TT}-2G_{Tt}+G_{tt}\right)}^{2} \end{array}$$

### Allelic case-control test

The simplest form of association test is the allelic case-control test based on the expressions for trait allele frequencies in cases and controls. The frequency of allele *T* among cases is as follows:

$$\begin{array}{c} p_{T|\mathrm{Case}}=\mathrm{Pr}\left(TT|\mathrm{Case}\right)+\frac{1}{2}\mathrm{Pr}\left(Tt|\mathrm{Case}\right)\\ =\left[\mathrm{Pr}\left(TT\;\mathrm{and}\;\mathrm{Case}\right)+\frac{1}{2}\mathrm{Pr}\left(Tt\;\mathrm{and}\;\mathrm{Case}\right)\right]/\mathrm{Pr}\left(\mathrm{Case}\right)\\ =\frac{p_{T}}{\mu_{G}}\left(p_{T}G_{TT}+p_{t}G_{Tt}\right) \end{array}$$

Similarly,

$$p_{T|\mathrm{Control}}=\frac{p_{T}}{1-\mu_{G}}\left[p_{T}\left(1-G_{TT}\right)+p_{t}\left(1-G_{Tt}\right)\right]$$

so that

$$p_{T|\mathrm{Case}}-p_{T|\mathrm{Control}}=\frac{p_{T}p_{t}}{\mu_{G}\left(1-\mu_{G}\right)}\left[p_{T}\left(G_{TT}-G_{Tt}\right)+p_{t}\left(G_{Tt}-G_{tt}\right)\right]$$

The square of this allelic frequency difference can be written as

$${\left(p_{T|\mathrm{Case}}-p_{T|\mathrm{Control}}\right)}^{2}=\frac{p_{T}p_{t}\sigma_{A_{G}}^{2}}{2{\left[\mu_{G}\left(1-\mu_{G}\right)\right]}^{2}}$$

The case-control test uses the sample allele frequencies ${\tilde{p}}_{T|\mathrm{Case}}$ and ${\tilde{p}}_{T|\mathrm{Control}}$ taken from independent samples of sizes *n*_Case_ and *n*_Control_. Still assuming HWE, these two sample values have binomial variances

$$\mathrm{Var}\left({\tilde{p}}_{T|\mathrm{Case}}\right)=\frac{1}{2n_{\mathrm{Case}}}p_{T|\mathrm{Case}}p_{t|\mathrm{Case}}$$

$$\mathrm{Var}\left({\tilde{p}}_{T|\mathrm{Control}}\right)=\frac{1}{2n_{\mathrm{Control}}}p_{T|\mathrm{Control}}p_{t|\mathrm{Control}}$$

and a test statistic is constructed under the null hypothesis that *p_T_*_|Case_ = *p_T_*_|Control_ = *p_T_*:

$$\begin{array}{c} z_{1}^{2}=\frac{{\left({\tilde{p}}_{T|\mathrm{Case}}-{\tilde{p}}_{T|\mathrm{Control}}\right)}^{2}}{\mathrm{Var}\left({\tilde{p}}_{T|\mathrm{Case}}-{\tilde{p}}_{T|\mathrm{Control}}\right)}\\ =\frac{2n_{\mathrm{Case}}n_{\mathrm{Control}}{\left({\tilde{p}}_{T|\mathrm{Case}}-{\tilde{p}}_{T|\mathrm{Control}}\right)}^{2}}{\left(n_{\mathrm{Case}}+n_{\mathrm{Control}}\right){\tilde{p}}_{T}{\tilde{p}}_{T}} \end{array}$$

where ${\tilde{p}}_{T}$ is an appropriate estimate of the population allele frequency *p_T_* . This estimate could be the pooled sample value:

$${\tilde{p}}_{T}=\frac{n_{\mathrm{Case}}{\tilde{p}}_{T|\mathrm{Case}}+n_{\mathrm{Control}}{\tilde{p}}_{T|\mathrm{Control}}}{n_{\mathrm{case}}+n_{\mathrm{Control}}}$$

or pooled according to disease prevalence

$${\tilde{p}}_{T}=\mu_{G}{\tilde{p}}_{T|\mathrm{Case}}+\left(1-\mu_{G}\right){\tilde{p}}_{T|\mathrm{Control}}$$

Power calculations do not assume the null hypothesis, and the parametric form of the test statistic cannot assume *p_T_*_|Case_ = *p_T_*_|Control_ = *p_T_*. As an approximation, however, this assumption could be retained in the denominator of the statistic, which then has noncentrality

$$\begin{array}{c} \lambda_{1}=\frac{2n_{\mathrm{Case}}n_{\mathrm{Control}}}{\left(n_{\mathrm{Case}}+n_{\mathrm{Control}}\right)}\frac{{\left(p_{T|\mathrm{Case}}-p_{T|\mathrm{Control}}\right)}^{2}}{p_{T}\left(1-p_{T}\right)}\\ =\frac{n\varphi \left(1-\varphi \right)\sigma_{A_{G}}^{2}}{{\left[\mu_{G}\left(1-\mu_{G}\right)\right]}^{2}} \end{array}$$

It is usual to use [(*n*_Case_*p_T_*_|Case_ + *n*_Control_*p_T_*_|Control_)/(*n*_Case_ + *n*_Control_)] for the population allele frequency *p_T_* in the denominator, even though it is [*μ_G_p_T_*_|Case_ + (1 − *μ_G_*)*p_T_*_|Control_] that is equal to *p_T_*, and the latter is the approach used in the present power calculations. Also, *n*_Case_ = *φn*, *n*_Control_ = (1 − *φ*)*n* for a total sample size of *n*.

The test statistic $z_{1}^{2}$ is distributed as χ2 with 1 df under the assumption of normally-distributed sample allele frequencies. This assumption will break down as frequencies become close to zero or one. We used simulation to assess the accuracy of power estimation at low MAF. Empirical power was estimated from 1,000,000 replicate simulations of data for each of several parameter sets, using the $z_{1}^{2}$ test and *α* = 5 × 10^−8^, whereas theoretical power was estimated using *λ*_1_. The parameter sets consist of all combinations of *GRR* ∈ {1.1, 1.2, 1.3, 1.4}, *N_cases_* = *N_controls_* ∈ {1000, 5000, 10000}, allelic frequency ∈ {0.005, 0.01, 0.02, 0.03, 0.04, 0.05}, and population prevalence of 0.05. Figure S10 shows that the two power estimates are very similar down to MAF of 0.02, but the theoretical calculations underestimate power at lower MAF. However, both power estimates are very small with the range of realistic parameter sets used here, so that this difference is probably not of practical importance in designing experiments.

### Allelic dosage test

Now suppose that each individual has an imputed genotype: the probabilities of it being imputed as *TT*, *Tt*, and *tt* are *Q_TT_*, *Q_Tt_*, and *Q_tt_* and the imputed allelic dosage is *q_T_* = *Q_TT_* + *Q_Tt_*/2. An association test with the trait can be constructed with sample allelic dosages in place of sample allelic frequencies:

$$z_{2}^{2}=\frac{{\left({\tilde{q}}_{T|\mathrm{Case}}-{\tilde{q}}_{T|\mathrm{Control}}\right)}^{2}}{\mathrm{Var}\left({\tilde{q}}_{T|\mathrm{Case}}-{\tilde{q}}_{T|\mathrm{Control}}\right)}$$

There is no reason to assume the imputed genotypes should follow HWE or that they depend in any specific way on the actual genotypes. For variants for which both observed genotypes and imputed allelic dosages are available, and assuming a linear relationship, imputed dosage can be regressed on observed allelic dosage. If the estimated regression coefficient $\hat{\beta }$ is then assumed to hold for subsequent samples of cases and controls:

$$\left({\tilde{q}}_{T|\mathrm{Case}}-{\tilde{q}}_{T|\mathrm{Control}}\right)=\hat{\beta }\left({\tilde{p}}_{T|\mathrm{Case}}-{\tilde{p}}_{T|\mathrm{Control}}\right)$$

Keeping the earlier assumptions about the variances of the allelic dosages being the same in cases and controls, and invoking the relations among regression coefficient *β_YX_*, correlation coefficient *ρ_XY_*, covariance *σ_XY_* and variances $\sigma_{X}^{2}$, $\sigma_{Y}^{2}$ for two variables *X*, *Y* it follows that

$$\sigma_{Y}^{2}=\frac{\sigma_{XY}^{2}}{\sigma_{X}^{4}}\times \frac{\sigma_{X}^{2}\sigma_{Y}^{2}}{\sigma_{XY}^{2}}\times \sigma_{X}^{2}=\frac{\beta_{YX}^{2}}{\rho_{XY}^{2}}\times \sigma_{X}^{2}$$

so that

$$\text{Var}\left({\tilde{q}}_{T|\mathrm{Case}}-{\tilde{q}}_{T|\mathrm{Control}}\right)=\frac{\beta^{2}}{\rho^{2}}\text{Var}\left({\tilde{p}}_{T|\mathrm{Case}}-{\tilde{p}}_{T|\mathrm{Control}}\right)$$

The noncentrality parameter for the test statistic becomes

$$\begin{array}{c} \lambda_{2}=\frac{\beta^{2}{\left(p_{T|\mathrm{Case}}-p_{T|\mathrm{Control}}\right)}^{2}}{\frac{\beta^{2}}{\rho^{2}}\left(\frac{1}{2n_{\mathrm{Case}}}+\frac{1}{2n_{\mathrm{Control}}}\right)p_{T}p_{t}}\\ =\rho^{2}\lambda_{1} \end{array}$$

where *ρ* is the correlation of observed and imputed allelic dosage. The effect of imputation is to decrease the total sample size by *ρ*^2^.
