# Supplementary material for: Imputation-Based Genomic Coverage Assessments of Current Human Genotyping Arrays
Source: G3 (Bethesda). 2013 Oct 1;3(10):1795–807. doi: 10.1534/g3.113.007161 (PMC3789804; doi:10.1534/g3.113.007161)
Supplement: Supporting Information [file supp_3_10_1795__index.html]

Imputation-Based Genomic Coverage Assessments of Current Human Genotyping Arrays — Supporting Information 

# Imputation-Based Genomic Coverage Assessments of Current Human Genotyping Arrays

## Supporting Information for Nelson *et al.*, 2013

**Files in this Data Supplement:**

- Supporting Information - File S1 and Figures S1-S10 (PDF, 1.4 MB)
- File S1 - Methods (PDF, 347 KB)
- Figure S1 - Mean imputation *r2*, by MAF bin and ancestry group (PDF, 460 KB)
- Figure S2 - Mean genotype concordance, by MAF bin and ancestry group (PDF, 352 KB)
- Figure S3 - Barplots of each of the four metrics summaries for AFR ancestry (PDF, 111 KB)
- Figure S4 - Barplots of each of the four metrics summaries for AMR ancestry (PDF, 111 KB)
- Figure S5 - Barplots of each of the four metrics summaries for ASN ancestry (PDF, 111 KB)
- Figure S6 - Barplots of each of the four metrics summaries for EUR ancestry (PDF, 111 KB)
- Figure S7 - Genome-wide power estimates for GRR values of 1.2, 1.3 and 1.4, for variants with MAF>0.01 (PDF, 178 KB)
- Figure S8 - Genome-wide power estimates for GRR values of 1.2, 1.3 and 1.4, for all variants with at least two copies of the minor allele seen in the given ancestry group (PDF, 555 KB)
- Figure S9 - Genome-wide power estimates for GRR=1.1, across MAF bins and ancestries (PDF, 618 KB)
- Figure S10 - Comparison of theoretical and empirical power of allelic association tests with low MAF (PDF, 339 KB)
