# Supplementary material for: Imputation-Based Genomic Coverage Assessments of Current Human Genotyping Arrays
Source: G3 (Bethesda). 2013 Oct 1;3(10):1795–807. doi: 10.1534/g3.113.007161 (PMC3789804; doi:10.1534/g3.113.007161)
Supplement: Supporting Information [file supp_g3.113.007161_FileS1.pdf]

File S1

Supporting Information: Methods

Here we give a more detailed description of the methods used to estimate genomic coverage.

Data Formatting:

We assessed genomic coverage using the publicly available 1000 Genomes Project (Abecasis *et al*., 2012) phase 1 integrated variant set, in variant call format (VCF, available at ftp://ftp.1000genomes.ebi.ac.uk/vol1/ftp/release/20110521/). The Project performed low-coverage whole genome sequencing and high-coverage targeted exome sequencing in 1,092 samples from fourteen populations worldwide. Samples have previously been grouped according to the "predominant component of ancestry:" African ("AFR"), American ("AMR"), Asian ("ASN"), and European ("EUR"; see Table 1) (Abecasis *et al.*, 2012). We first randomly assigned each of the 1,092 1000 Genomes samples into one of ten batches, balancing samples across ancestry group and population. Nine of the ten batches contained 110 samples; the remaining batch contained 102 samples.

We used vcftools software (Danecek *et al*., 2011) to create subsets of the data in the 1000 Genomes VCF files, which are separated by chromosome. For each sample batch, we extracted genotypes from the VCF files for just the samples in the batch and the variants on the array and converted into PLINK-formatted datasets (Purcell *et al*., 2007). With vcftools conversion options, only bi-allelic variants are retained when reformatting from VCF to PLINK, such that variants with more than one non-reference allele were dropped during this initial formatting step. Array variants were identified within the 1000 Genomes VCF using chromosome and base pair positions from the vendor-supplied array manifests. Where more than one 1000 Genomes (bi-allelic) variant occurred at the same positions as an array variant [i.e. 1000 Genomes data included both a single nucleotide variant (SNV) and an insertion/deletion variant at a given position], we retained only the first variant. This removal of duplicated positions was to comply with requirements of the pre-phasing software that each position be unique.

Pre-phasing and imputation:

Pre-phasing has been widely adopted as a way to maintain imputation accuracy while minimizing computation time, as available reference panels increase in number and in size (Howie *et al.*, 2012). The computational arguments for pre-phasing are that (1) imputing into pre-phased haplotypes is much faster than imputing into unphased genotypes and (2) pre-phased data facilitates future updates to imputation, as improved reference panels become available. Although pre-phasing may introduce a small loss of accuracy, due to the lack of incorporating haplotype uncertainty information into the imputation step, the advantages appear to outweigh the disadvantages for most genome-wide association studies (GWAS). Thus we adopted a

two-stage pre-phasing and imputation approach for these coverage analyses to estimate the coverage a typical GWAS would get from a pre-phased imputation.

The 1000 Genomes VCF files contain mostly phased genotypes. However, to better approximate array genotypes, which are usually at first unphased, we discarded phasing information when converting from VCF to PLINK. We then used SHAPEIT2 (Delaneau *et al.*, 2013) software to pre-phase each dataset. The resulting phased haplotypes were then imputed with IMPUTE2 (Howie *et al.*, 2011) where, for each batch, the remaining 1000 Genomes samples served as a worldwide imputation reference panel. The reference panel data were downloaded from the IMPUTE2 website (http://mathgen.stats.ox.ac.uk/impute/impute_v2.html#reference), which contains IMPUTE2-formatted versions of the 1000 Genomes phase 1 VCF files. We restricted imputation to variants with at least two copies of the minor allele in any one of the four ancestry groups, yielding ~30.3 million variants across the autosomes and non-pseudoautosomal regions of chromosome X (out of over 39 million in the full 1000 Genomes dataset). Following imputation, the output (genotype probabilities file) for each of the ten sample batches was then combined.

Metrics calculation:

To assess imputation accuracy and by extension genomic coverage, we compared imputed results at all the non-array variants to observed genotypes from the original 1000 Genomes VCF files. These comparisons were performed separately by ancestry group and restricted to variants with at least two copies of the minor allele in the given ancestry group, yielding 22,562,947 variants in the AFR group; 14,994,964 in AMR; 11,610,609 in ASN; and 13,696,676 in EUR. This variant restriction was done to avoid missing imputation metrics, which occur when a variant is either imputed or observed to be monomorphic. Imputation included all three variant types in this 1000 Genomes Project release: single nucleotide, insertion/deletion, and structural variants, and thus all three variant types were included in these metrics calculations. Calculations were coded in the R statistical and graphing software environment (http://www.r-project.org/), and made use of the VariantAnnotation package (Obenchain *et al.*, 2013) for reading in the VCF files.

At each imputed variant we calculated three metrics: (1) the squared correlation between observed and imputed allelic dosages, which we call “imputation r^2;” (2) the concordance between observed and most likely imputed genotypes, the “genotype concordance;” and (3) the concordance between observed and most likely imputed genotypes, when at least one of those two genotypes contains one or two copies of the minor allele, which we call “minor allele (MA) concordance.” Array (observed) variants are included in these metrics summaries and are given imputation r^2, genotype concordance, and MA concordance values of 1. Both imputed and observed variants were grouped into four bins by minor allele frequency (MAF) in the given ancestry group: MAF≤0.01 (but with at least two copies of the minor allele), 0.01<MAF≤0.05, MAF>0.01, and

MAF>0.05. We summarized metrics across variants in each of these MAF bins in four ways: (1) the fraction of variants with imputation $r^2 \geq 0.8$, (2) the mean imputation r^2, (3) the mean genotype concordance, and (4) the mean MA concordance.

STUDY LIMITATIONS

Several factors may result in our genomic coverage estimates either under or overestimating the true coverage. In the following discussion, we highlight some of these factors; state whether we expect them to result in under or overestimation; and, where possible, suggest remedies or at least ways of interpreting our results given these limitations.

Firstly, by assessing genomic coverage in the context of genome-wide imputation, we can only assess array variants that are also present in our chosen reference panel (1000 Genomes phase 1 integrated variant set). Thus not all array variants can inform the imputation, for one of two reasons: (1) the array variant is not present in this 1000 Genomes dataset or (2) the array variant is present but without two or more copies of the minor allele in any one of the four panels (our filtering threshold for imputation; see Table 2). The 1000 Genomes Project set out to capture variants with MAF>0.01 (Durbin *et al*., 2010). Thus, array content focused on rare variants and other specialized content (e.g., exome and pharmacogenetic variants) is less likely to be found in our chosen 1000 Genomes reference compared to more common array variants. In addition, the metrics summaries for each ancestry group following imputation only include variants with at least two copies of the minor allele in the given ancestry group. Thus array variants could also drop out of the analysis at the point of summarizing and plotting the quality metrics.

Thus this approach could be seen as giving preferential treatment to the arrays focusing on common variation to the exclusion of rare variation. Previous genomic coverage analyses (Li *et al.*, 2008) have also noted this issue and that it may yield an underestimated genomic coverage. We apply a suggested correction for handling of these non-reference variants below. Nonetheless, our approach is an accurate reflection of an imputation that may follow genotyping with one of these arrays. That is, one can only impute using those array variants also found in the reference panel. The remaining array content is undoubtedly of interest for other applications, but not imputation as we have performed it here.

Different genomic coverage equations:

When quantifying genomic coverage as the fraction of variants passing a imputation $r^2 \geq 0.8$, we have used the equation (L+T)/R, where T is the count of variants directly genotyped on the array; L is the count of variants indirectly captured by T (i.e. with imputation $r^2 \geq 0.8$); and R is the count of reference variants. In our application of the formula, R is defined as all variants in the 1000 Genomes Project phase 1 integrated release set having at least two copies of the minor allele in the given ancestry group. When this equation was first outlined by Barrett and Cardon (2006), they noted at least two ways in which the estimate may be “naïve” and in need of refinement. Firstly, R is only a subset of all the variants in the genome, G. This was especially true when

R was a set of HapMap single nucleotide polymorphisms (SNPs). Barrett and Cardon proposed a correction to the naïve equation that requires an estimate of G, which they drew from the ENCODE project. In order to reasonably estimate G one needs to set a lower bound on MAF, which Barrett and Cardon set at 0.05. To date, each newly sequenced human genome has uncovered novel variants (Zhang *et al.*, 2011), thus we cannot hope to assess coverage across the totality of variants in the human genome. The 1000 Genomes Project has captured 98% of all variants with MAF of 0.01 and 99.7% of all variants with MAF of 0.05 (Abecasis *et al.*, 2012). Thus, here when we have set the lower bound of MAF at 0.01, R becomes very close to G and the original formula of (L+T)/R is less "naïve."

In the formulation (L+T)/R, T is limited to the set of array SNPs also overlapping with the reference set R. Thus a second refinement may be needed to account for array SNPs not in the reference panel, which has been outlined by Li *et al*. (2008). In brief, the count of non-reference SNPs on the array *m* can be added to both the reference set (R_1=R+*m*) and the array SNPs (T_1=T+*m*). One then wants to estimate L_1, or the set of variants covered by all array variants, including the ones not in the reference set (*m).* Assuming that the number of variants indirectly covered increases proportionally to the directly genotyped variants, then $L_1 \approx (T_1/T) \times L$. Then the adjusted estimate of genomic coverage becomes $[L_1/(R_1 - T_1) \times (G - T_1) + T_1]/G$. Similarly to the original formula, this estimate is also applied to a certain MAF level (e.g., all common SNPs, or MAF>0.05). Thus in order to apply this formula, one needs to know the MAF of non-reference array variants in order to correctly assign them to the set *m*.

In order to determine the effect of applying this adjustment to our genomic coverage analyses, we took a test case of the Omni2.5M array in the EUR ancestry group. While only 93% of array positions occur in the 1000 Genomes reference panel used here, the Project has performed Omni2.5M genotyping on 2,123 samples (including the 1,092 in the phase 1 integrated release; see ftp://ftp.1000genomes.ebi.ac.uk/vol1/ftp/technical/working/20111117_omni_genotypes_and_intensities/). Thus we used this separately genotyped set to determine the EUR MAF of non-reference array variants. Of the array variants not in the 1000 Genomes reference set, 36,183 had a MAF greater than 0.01 in EUR samples, providing a value for *m*. Because R_1 (R+*m*) was now greater than R, we increased G to R/0.98, as phase 1 of the 1000 Genomes Project includes 98% of all variants with MAF≈0.01.

With this corrected formula, we obtained a coverage estimate of 82.97% for the EUR panel on the Omni2.5M, compared to our initial value of 81.45% (see Table 4). We repeated the calculation for the AFR, AMR, and ASN panels and found the adjusted genomic coverage values to be consistently ~1.5% higher compared to our initial, unadjusted values. These findings indicate that genomic coverage values using the formula (L+T)/R may be slightly underestimated. The situation with the Omni2.5M array is unique in that 1000 Genomes samples have been genotyped on this array, and thus we can calculate panel-specific MAF for array variants not in the phase 1 release. This exercise is necessary to quantify the *m* needed to apply the Li *et*

al. adjustment. However, to our knowledge the Project has not released genotype information for these other arrays and thus we cannot calculate the panel-specific MAF values needed to quantify *m*. Thus we take this example of applying the adjusted formula to the Omni2.5M to suggest that coverage of other arrays may be similarly underestimated. Users should note the degree of overlap between arrays and the 1000 Genome phase 1 release presented in Table 2 and be aware that the coverage we present for arrays with less overlap (e.g., the HumanCore+Exome and Affymetrix Biobank) may be more of an underestimate than for the Omni2.5M.

Reference panel:

An additional limitation, which distinguishes our analyses from more real world scenarios, is that our study samples and reference panel are derived from the same dataset – i.e. the 1000 Genomes Project. In reality, the study samples would be drawn from different populations and thus the representativeness of the reference panel would likely be less than what we have done here. This overestimated representativeness, however, is equal across the different arrays in our comparison. Thus while our imputation quality metrics for each array may be biased upwards in absolute terms, they can safely be compared relative to the other arrays. However, we also removed the test samples from the reference panel while imputing each of the ten test batches. Thus our reference panel contained either 982 (for the first nine batches) or 990 (for the last batch) samples, rather than the full set of 1,092 samples. Imputation accuracy has been shown to increase with increasing reference panel size (Browning and Browning, 2009; Howie *et al.*, 2011). Thus the overestimate of coverage we may get by using study samples derived from the same populations as the reference panel may be counteracted somewhat by the decrease in accuracy caused by having a smaller reference panel.

An additional way that our use of the 1000 Genomes Project as reference panel may result in overestimated genomic coverage is when data from the Project informed array design. As noted by Barrett and Cardon (2006), “coverage estimates will be biased upward if all or part of the reference set used for the estimate was used to select tag SNPs.” We surveyed product documentation and any other available literature (e.g., journal articles) to determine which of these eight arrays was designed using 1000 Genomes data. We found evidence that content selection for the majority of arrays was in some way guided by 1000 Genomes Project pilot or phase 1 data.

Of the Illumina arrays, the Omni2.5M and Omni2.5M+Exome were designed using data from the 1000 Genomes pilot project (Illumina, 2013). Design of the Omni5M was informed by phase 1 of the Project (Dec. 2012 release) (Illumina, 2013). Content selection for the OmniExpress was based on all three phases of the HapMap Project rather than 1000 Genomes, although there is sample overlap between the two. Tagging content for the HumanCore array was mostly drawn from the OmniExpress (and thus HapMap), although a modest number of SNPs (~5,000) were selected based on their presence in 1000

Genomes Project data (D. Bullis, Illumina Product Manager, personal communication, March 29 and May 22, 2013). The most detailed information available was for the Axiom World Array 4, which used 1000 Genomes (phase 1 interim release) data to maximize genome-wide coverage of variants in Latino populations (Hoffmann *et al*., 2011). The tagging content of the Axiom Biobank array was selected via the same imputation-based genomic coverage approach as the World Arrays, and thus is also partially dependent on 1000 Genomes Project data (Affymetrix, 2012). Thus for most of the arrays we have assessed, coverage estimates may be slightly inflated due to the circular nature of the assessment: tagging content initially selected based on 1000 Genomes data was subsequently assessed for coverage via 1000 Genomes data (albeit potentially different releases: pilot, phase 1 interim, or phase 1 integrated). However, because phase 1 of the 1000 Genomes Project contains ~98% of all SNPs with frequency ~0.01 (Abecasis *et al.*, 2012), we expect this bias to be small.

Summary

The actual coverage achieved by any of these arrays in a “real-world” dataset will be affected by numerous study-specific factors, such as size and ethnic composition of the dataset, genotyping error rate, and the settings used in both pre-phasing and imputation software programs. However, our coverage estimates are internally consistent in that the same method was used to pre-phase, impute, and analyze each array. Thus we expect these analyses to enable researchers to objectively compare coverage across different arrays in different ancestry groups.

References

Abecasis, G. R., Auton, A., Brooks, L. D., DePristo, M. A., Durbin, R. M., Handsaker, R. E., et al. (2012). An integrated map of genetic variation from 1,092 human genomes. *Nature, 491*(7422), 56-65.

Affymetrix. (2012). Axiom® Biobank Genotyping Arrays (Data Sheet). Retrieved March 4, 2013, from http://media.affymetrix.com/support/technical/datasheets/axiom_biobank_genotyping_arrays_datasheet.pdf

Barrettt, J. C., & Cardon, L. R. (2006). Evaluating coverage of genome-wide association studies. *Nat Genet, 38*(6), 659-662.

Browning, B., & Browning, S. (2009). A unified approach to genotype imputation and haplotype-phase inference for large data sets of trios and unrelated individuals. *Am J Hum Genet, 84*(2), 210-223.

Danecek, P., Auton, A., Abecasis, G., Albers, C. A., Banks, E., DePristo, M. A., et al. (2011). The variant call format and VCFtools. *Bioinformatics, 27*(15), 2156-2158.

Delaneau, O., Zagury, J. F., & Marchini, J. (2013). Improved whole-chromosome phasing for disease and population genetic studies. *Nat Methods, 10*(1), 5-6.

Durbin, R. M., Abecasis, G. R., Altshuler, D. L., Auton, A., Brooks, L. D., Gibbs, R. A., et al. (2010). A map of human genome variation from population-scale sequencing. *Nature, 467*(7319), 1061-1073.

Hoffmann, T. J., Zhan, Y., Kvale, M. N., Hesselson, S. E., Gollub, J., Iribarren, C., et al. (2011). Design and coverage of high throughput genotyping arrays optimized for individuals of East Asian, African American, and Latino race/ethnicity using imputation and a novel hybrid SNP selection algorithm. *Genomics, 98*(6), 422-430.

Howie, B., Fuchsberger, C., Stephens, M., Marchini, J., & Abecasis, G. R. (2012). Fast and accurate genotype imputation in genome-wide association studies through pre-phasing. *Nat Genet, 44*(8), 955-959.

Howie, B., Marchini, J., & Stephens, M. (2011). Genotype Imputation with Thousands of Genomes. *G3: Genes, Genomics, Genetics 1*(6), 457-470

Illumina, I. (2013). Omni Family of Arrays. Retrieved May 20, 2013, 2013, from http://www.illumina.com/applications/genotyping/omni_family.ilmn

Li, C., Li, M., Long, J. R., Cai, Q., & Zheng, W. (2008). Evaluating cost efficiency of SNP chips in genome-wide association studies. *Genet Epidemiol, 32*(5), 387-395.

Obenchain, V., Morgan, M., & Lawrence, M. (2013). VariantAnnotation: Annotation of Genetic Variants. R package version 1.4.7.

Purcell, S., Neale, B., Todd-Brown, K., Thomas, L., Ferreira, M., Bender, D., et al. (2007). PLINK: a tool set for whole-genome association and population-based linkage analyses. *Am J Hum Genet, 81*(3), 559-575.

Zhang, J., Chiodini, R., Badr, A., & Zhang, G. (2011). The impact of next-generation sequencing on genomics. *J Genet Genomics, 38*(3), 95-109.
