# Supplementary material for: Imputation-Based Genomic Coverage Assessments of Current Human Genotyping Arrays
Source: G3 (Bethesda). 2013 Oct 1;3(10):1795–807. doi: 10.1534/g3.113.007161 (PMC3789804; doi:10.1534/g3.113.007161)
Supplement: Supporting Information [file supp_g3.113.007161_FigureS1.pdf]

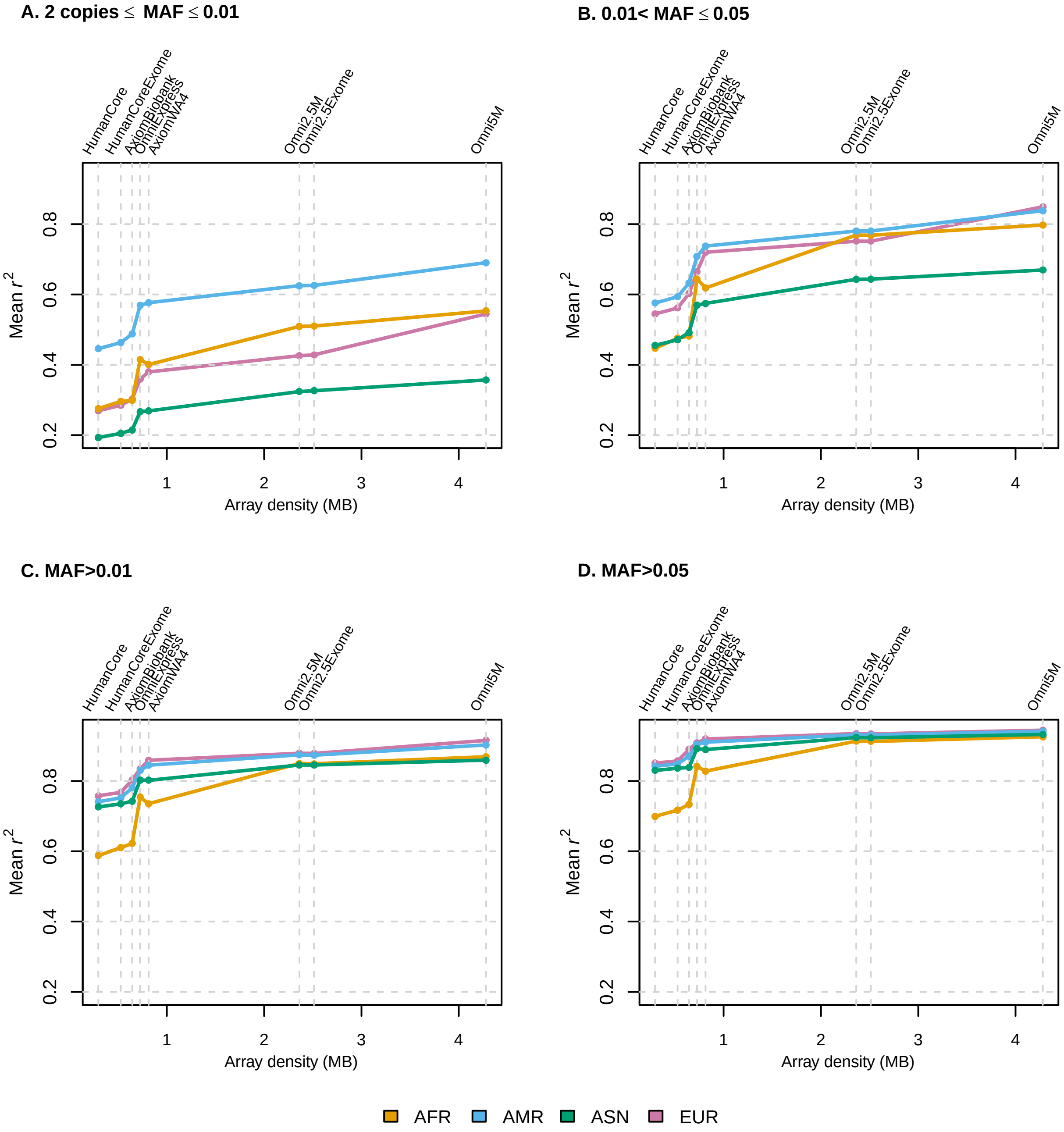

Figure S1 Mean imputation r^2, by MAF bin and ancestry group. Panel (A) is for variants with at least two copies of the minor allele and MAF≤0.01, (B) for 0.01<MAF≤0.05, (C) for MAF>0.01, and (D) for MAF>0.05.
