# Supplementary material for: Imputation-Based Genomic Coverage Assessments of Current Human Genotyping Arrays
Source: G3 (Bethesda). 2013 Oct 1;3(10):1795–807. doi: 10.1534/g3.113.007161 (PMC3789804; doi:10.1534/g3.113.007161)
Supplement: Supporting Information [file supp_g3.113.007161_FigureS3.pdf]

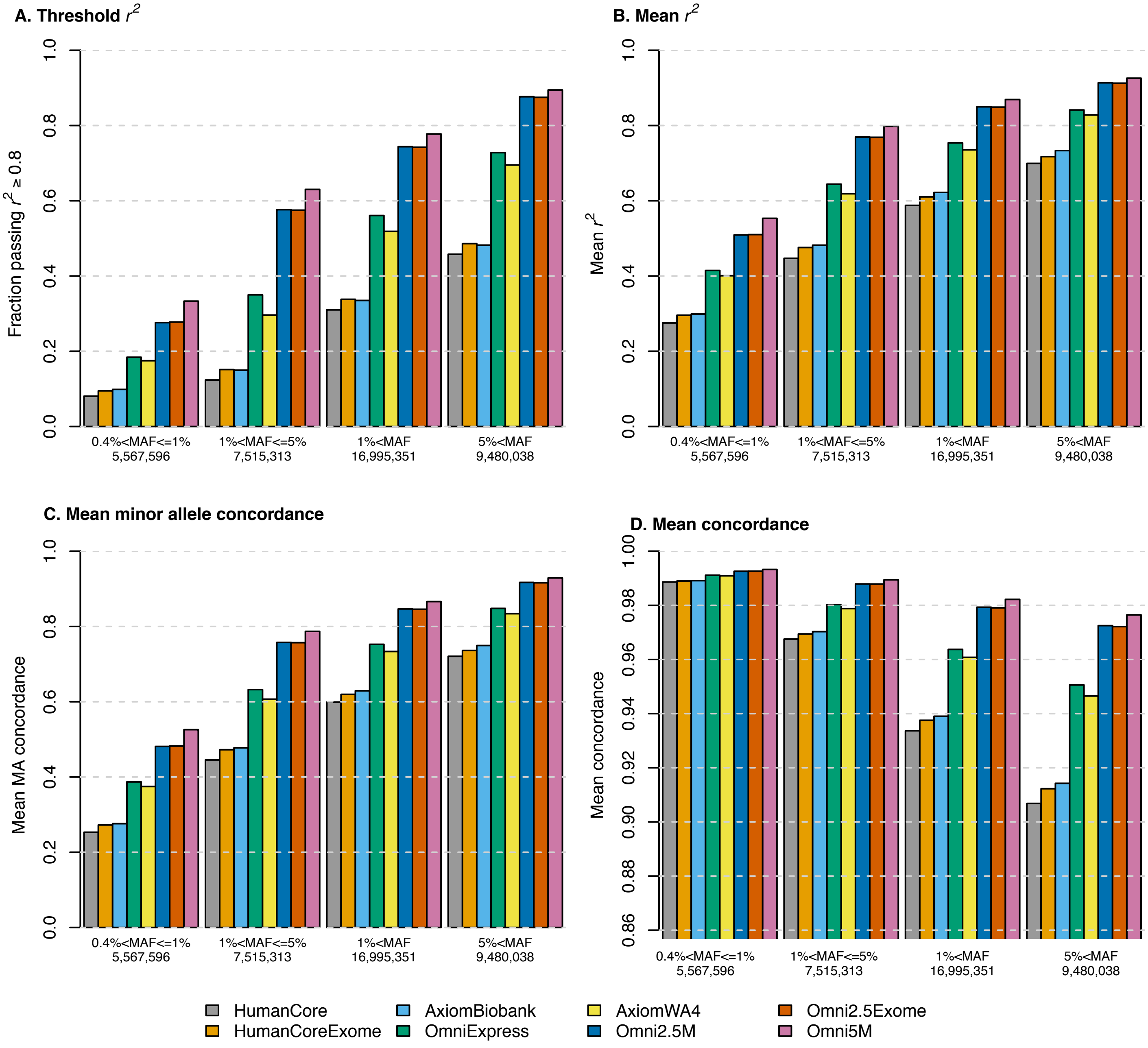

Figure S3 Barplots of each of the four metrics summaries for AFR ancestry. The x-axis indicates MAF bin, including variant count within each bin; the colors of the bars denote the array, which are ordered from left to right based on density. The data presented here are the same as shown elsewhere (line plots in Figures 2, 3, S1, and S2; and Table 3), but with a different visual organization. The fraction of variants with imputation $r^2 \geq 0.8$ is shown in the top left; mean imputation r^2 in the top right; mean minor allele concordance in the bottom left; and mean genotype concordance in the bottom right.
