# Supplementary material for: Imputation-Based Genomic Coverage Assessments of Current Human Genotyping Arrays
Source: G3 (Bethesda). 2013 Oct 1;3(10):1795–807. doi: 10.1534/g3.113.007161 (PMC3789804; doi:10.1534/g3.113.007161)
Supplement: Supporting Information [file supp_g3.113.007161_FigureS7.pdf]

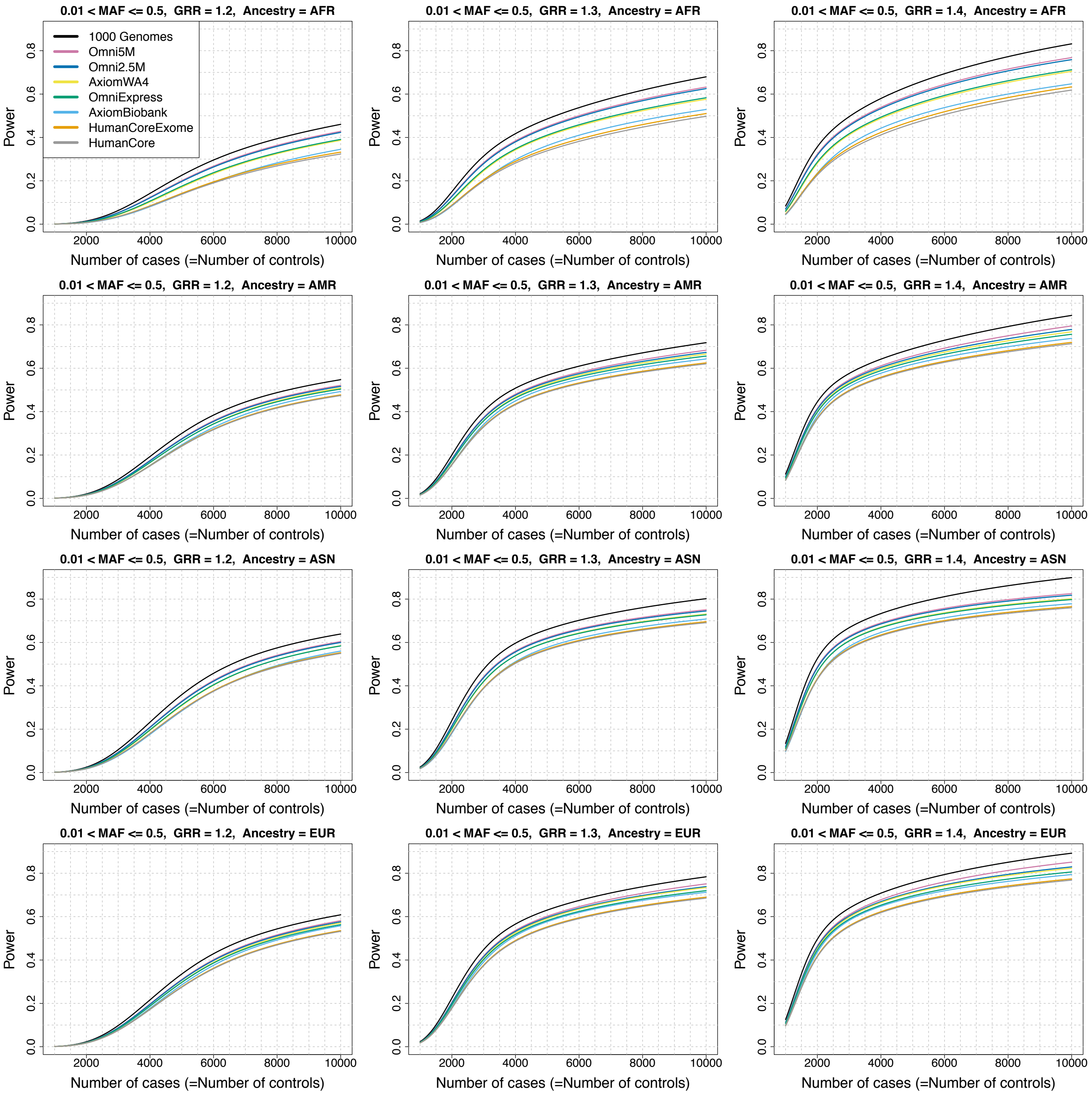

Figure S7 Genome-wide power estimates for GRR values of 1.2, 1.3 and 1.4, for variants with MAF>0.01. The array Omni2.5+Exome is not shown in these plots because it is indistinguishable at this resolution from the Omni2.5M array. In the legend, "1000 Genomes" refers to a hypothetical array in which all variants in the 1000 Genomes dataset would be typed.
