# Supplementary material for: Imputation-Based Genomic Coverage Assessments of Current Human Genotyping Arrays
Source: G3 (Bethesda). 2013 Oct 1;3(10):1795–807. doi: 10.1534/g3.113.007161 (PMC3789804; doi:10.1534/g3.113.007161)
Supplement: Supporting Information [file supp_g3.113.007161_FigureS8.pdf]

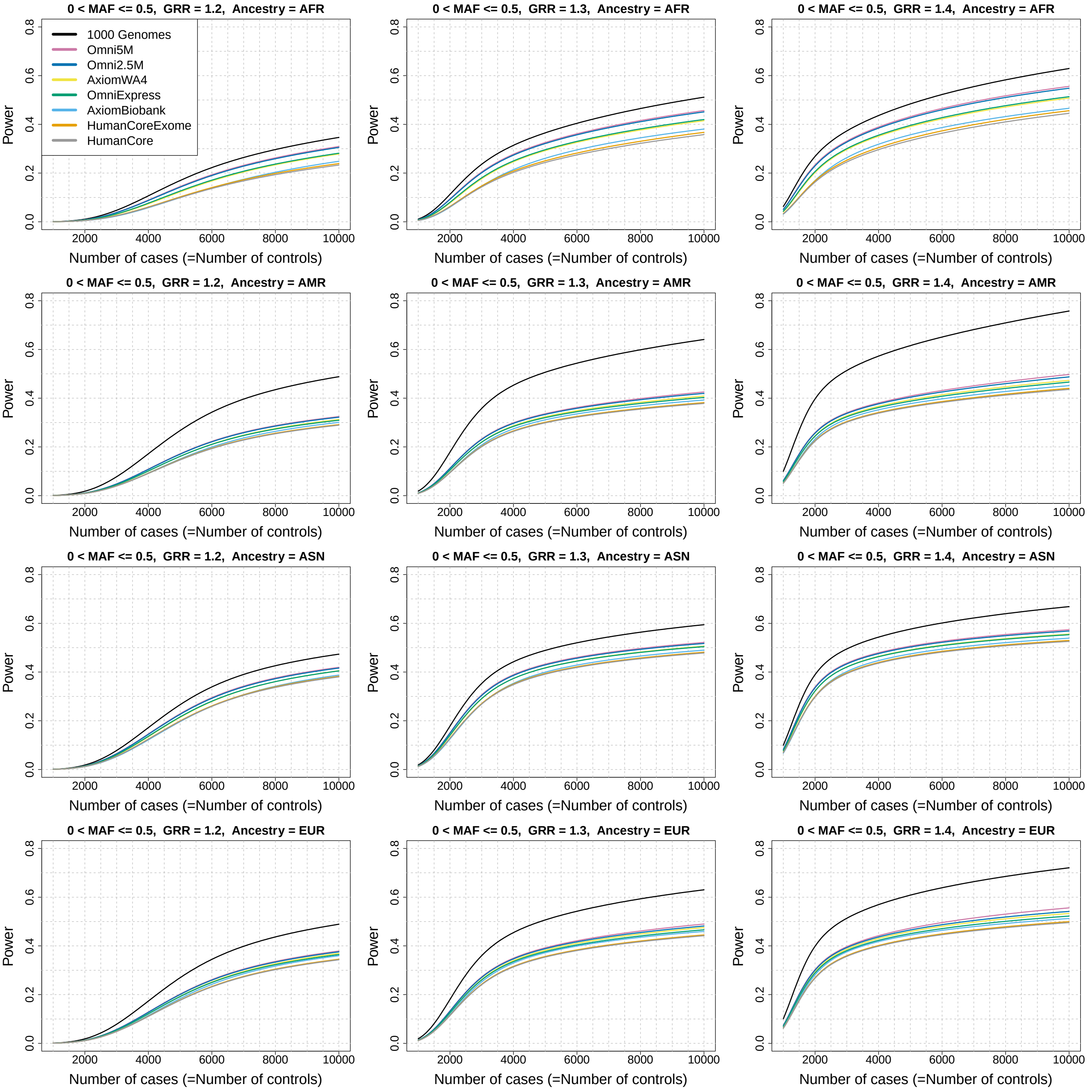

Figure S8 Genome-wide power estimates for GRR values of 1.2, 1.3 and 1.4, for all variants with at least two copies of the minor allele seen in the given ancestry group. The array Omni2.5+Exome is not shown in these plots because it is indistinguishable at this resolution from the Omni2.5M array. In the legend, “1000 Genomes” refers to a hypothetical array in which all variants in the 1000 Genomes dataset would be typed.
