# Supplementary material for: Imputation-Based Genomic Coverage Assessments of Current Human Genotyping Arrays
Source: G3 (Bethesda). 2013 Oct 1;3(10):1795–807. doi: 10.1534/g3.113.007161 (PMC3789804; doi:10.1534/g3.113.007161)
Supplement: Supporting Information [file supp_g3.113.007161_FigureS10.pdf]

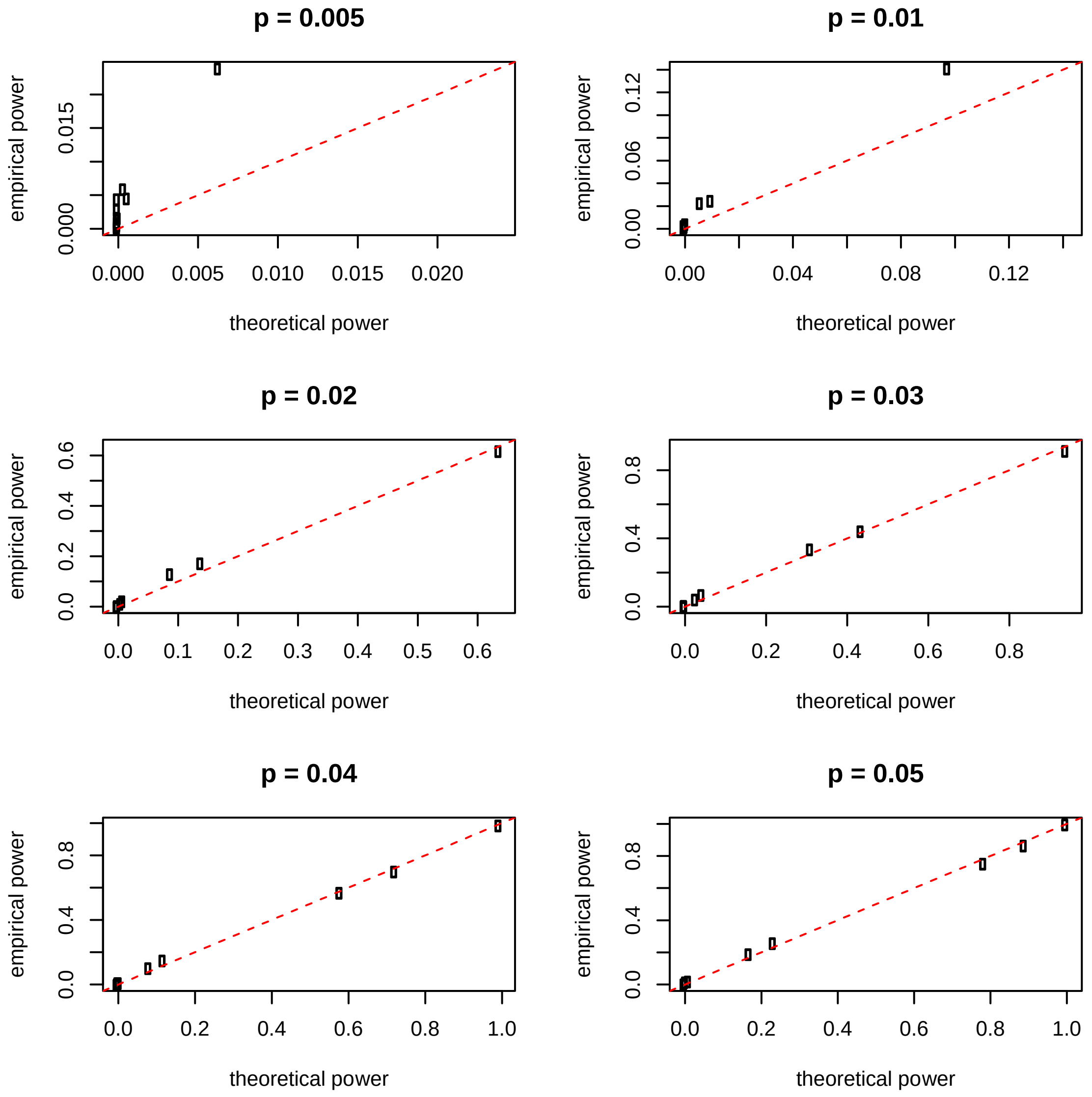


**Figure S10 Comparison of theoretical and empirical power of allelic association tests with low MAF.** Empirical power was estimated from 1,000,000 replicate simulations of data for each of several parameter sets, using the $z_1^2$ test and α = 5 x $10^{-8}$, while theoretical power was estimated using the non-centrality parameter $\lambda_1$ defined in the Appendix. The parameter sets consist of all combinations of *GRR* $\in$ {1.1,1.2,1.3,1.4}, $N_{cases} = N_{controls}$ $\in$ {1000,5000,10000}, population prevalence of 0.05, and allelic frequency *p* $\in$ {0.005,0.01,0.02,0.03,0.04,0.05}. Each plot is for a different MAF and each point represents the results for one parameter set.
